# Metal ions/nucleotide coordinated nanoparticles comprehensively suppress tumor by synergizing ferroptosis with energy metabolism interference

**DOI:** 10.1186/s12951-022-01405-w

**Published:** 2022-04-26

**Authors:** Yanqiu Wang, Jie Chen, Jianxiu Lu, Juqun Xi, Zhilong Xu, Lei Fan, Hua Dai, Lizeng Gao

**Affiliations:** 1 School of Medicine, Institute of Translational Medicine, Yangzhou University, Yangzhou, 225009 People’s Republic of China; 2 Jiangsu Key Laboratory of Integrated Traditional Chinese and Western Medicine for Prevention and Treatment of Senile Diseases, Yangzhou, 225009 People’s Republic of China; 3 School of Chemistry and Chemical Engineering, Yangzhou University, Yangzhou, 225002 People’s Republic of China; 4 CAS Engineering Laboratory for Nanozyme, Key Laboratory of Protein and Peptide Pharmaceutical Institute of Biophysics, Chinese Academy of Sciences, Beijing, 100101 People’s Republic of China

**Keywords:** Nano ferroptosis inducers, Metal ion–nucleotide interaction, GAPDH siRNA, Energy metabolic interference, Cancer synergistic therapy

## Abstract

**Background:**

Ferroptosis holds promise as a potential tumor therapy by programming cell death with a hallmark of reactive oxygen species (ROS)-induced lipid peroxidation. However, vigorous energy metabolism may assist tumors to resist oxidative damage and thus weaken the effects of ferroptosis in tumor treatment.

**Results:**

Herein, a bifunctional antitumor platform was constructed via coordinated interactions between metal ions and nucleotides to synergistically activate ferroptosis and interrupt energy metabolism for tumor therapy. The designed nanoparticles were composed of Fe^2+^/small interfering RNA (siRNA) as the core and polydopamine as the cloak, which responded to the tumor microenvironment with structural dissociation, thereby permitting tumor-specific Fe^2+^ and siRNA release. The over-loaded Fe^2+^ ions in the tumor cells then triggered ferroptosis, with hallmarks of lipid peroxidation and cellular glutathione peroxidase 4 (GPX4) down-regulation. Simultaneously, the released siRNA targeted and down-regulated glyceraldehyde-3-phosphate dehydrogenase (GAPDH) expression in the tumor to inhibit glycolytic pathway, which interfered with tumor energy metabolism and enhanced Fe^2+^-induced ferroptosis to kill tumor cells.

**Conclusions:**

This study presents a concise fabrication of a metal ion/nucleotide-based platform to integrate ferroptosis and energy metabolism intervention in one vehicle, thereby providing a promising combination modality for anticancer therapy.

**Graphical Abstract:**

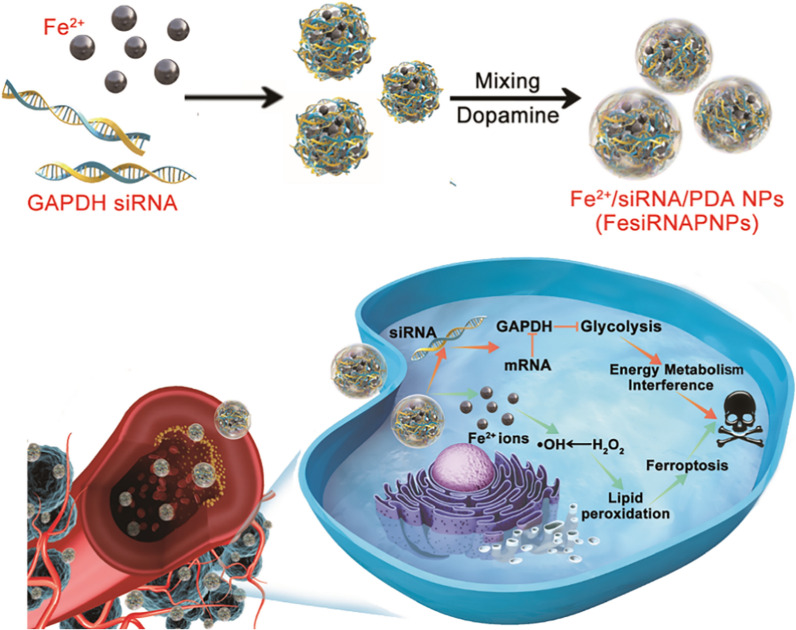

**Supplementary Information:**

The online version contains supplementary material available at 10.1186/s12951-022-01405-w.

## Background

Despite tremendous progress in antitumor research, tumor therapy remains a considerable clinical challenge [1, 2]. Conventional therapeutic modalities, such as radiotherapy and chemotherapy, inevitably cause adverse side effects in normal cells and tissues, with the sharp decline in their efficacy as a result of the emergence of resistance in tumor cells [3, 4]. Therefore, the development of new therapeutic strategies based on alternative mechanisms of tumor inhibition is urgently needed [5–7]. Ferroptosis is a newly discovered type of regulated cell death that involves intracellular iron accumulation and lipid peroxidation [8, 9]. This progress is distinct from necroptosis, apoptosis, and autophagic cell death, with great potential for cancer therapy. Recently, various ferroptosis-inducing agents have been designed [10]. Among these, nanoparticle-based inducers provide new choices in ferroptosis induction or sensitization, where multifunctional nano ferroptosis inducers can be fabricated based on their varied physicochemical properties [11, 12]. For example, iron (Fe)-based nanomaterials, such as PEGylated single-atom Fe nanocatalysts [13], pyrite (FeS_2_) nanoparticles [14], and Fe-organic frameworks [15], have been used as ferroptosis agents to trigger the Fenton reaction to up-regulate ROS levels, thus causing tumor cell death. Thus, interdisciplinary cooperation in materials science, chemistry, and cancer biology should boost the development of ferroptosis-related research for tumor therapy.

The main driving force of ferroptosis is the cellular ROS stress produced by energy metabolism [16]. Owing to the rapid growth of tumor cells and acceleration of their metabolic rates, ROS levels in tumor cells are usually enhanced compared with that in normal cells, rendering them less susceptible to ferroptosis [17]. Recent reports show that intracellular energy metabolism, such as pentose phosphate pathway, glycolysis, and tricarboxylic acid cycle, is directly related to ferroptosis via regulation of antioxidant defense [18–20]. For self-preservation, tumor cells are able to activate adaptive metabolic responses, i.e., up-regulation of glycolysis and pentose phosphate pathway, to inhibit ferroptosis [18–20]. Therefore, targeting important processes in energy metabolism of tumors may provide opportunities to promote the tumor susceptibility to ferroptosis and develop new and effective ferroptosis inducers for tumor therapies. Recently, small interfering RNA (siRNA) has been used as a promising strategy to regulate tumor signal pathways for cancer therapy [21–24]. In particular, to enhance siRNA delivery efficiency and avoid potential side effects, various non-viral nanosystems for delivering siRNAs have been developed, including micelles, emulsions, liposomes, and solid lipid nanoparticles [25–27]. Our hypothesis is that if a nanocarrier performs biological function (e.g. activating ferroptosis) in addition to delivering siRNA to regulate glycolytic pathway, it may achieve synergistic effects for tumor therapy.

**Scheme 1:**
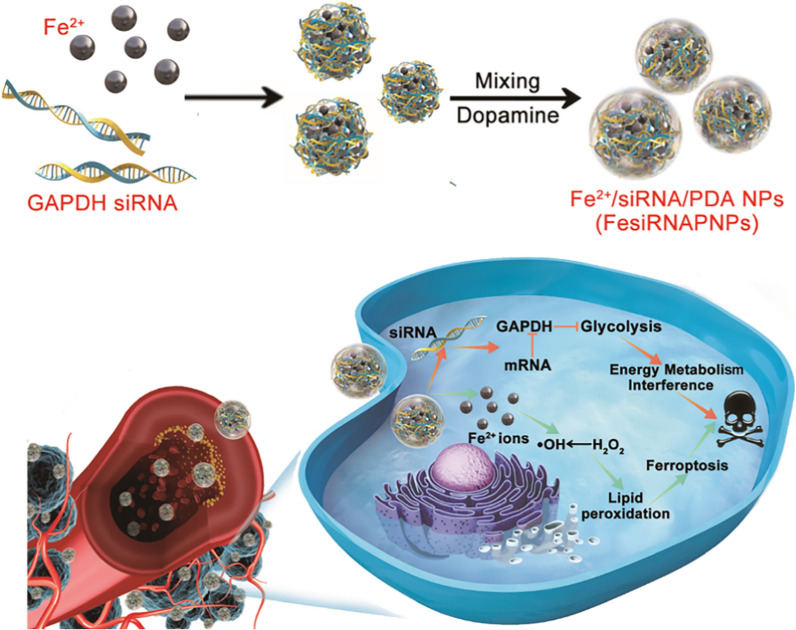
Schematic of FesiRNAPNPs synthesis and their antitumor activity by synergizing ferroptosis with energy metabolism interference

Here, we report on the facile construction of a new type of nano ferroptosis inducer via coordinated metal–ligand interactions. Guanosine monophosphate (GMP) was first chelated with ferrous ions and then immersed in dopamine-containing buffer solution to produce polydopamine (PDA), thus yielding Fe^2+^/GMP/PDA nanoparticles (FeGPNPs). The obtained FeGPNPs, as Fenton reaction catalysts, were able to transform endogenous H_2_O_2_ into highly toxic hydroxyl radicals (·OH). Treatment of cancer cells with FeGPNPs induced remarkably elevated intracellular Fe ion concentration, which then caused the accumulation of cytotoxic lipid hydroperoxides, leading to ferroptosis. Notably, this construction strategy could be explored due to the advantages of tunable types of metal ions and ligands. Considering the relationship between energy metabolism and ferroptosis, we used another nucleotide, glyceraldehyde-3-phosphate dehydrogenase (GAPDH) small interfering RNA (siRNA), to replace GMP to synthesis Fe^2+^/siRNA/PDA nanoparticles (FesiRNAPNPs). In the obtained FesiRNAPNPs, the GAPDH siRNA silenced the target mRNA to inhibit glycolysis, interfered with tumor energy metabolism, and enhanced Fe^2+^-induced ferroptosis (Scheme 1). A positive response to ferroptosis therapy with interference of energy metabolism was achieved in vitro and in vivo. Consequently, we developed a new nanotherapeutic strategy to enhance antitumor efficacy by synergizing ferroptosis with energy metabolism interference, which may bring forth new ideas for improving ferroptosis-based tumor therapies in the future.

## Results and discussion

**Fig. 1:**
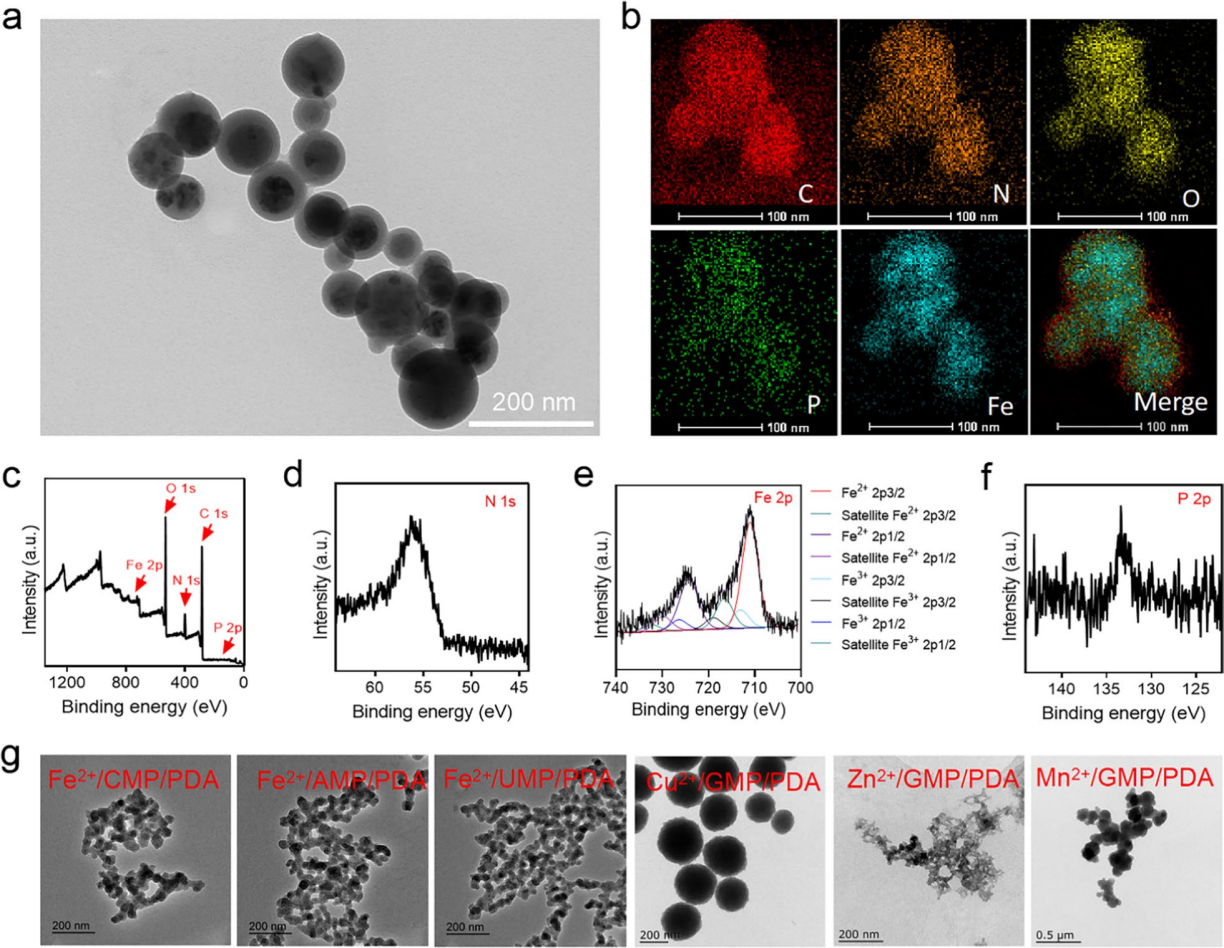
Characterization of FeGPNPs based on metal and nucleotide coordination. **a** TEM image of FeGPNPs. **b** EDX mapping of FeGPNPs. **c** Survey XPS spectrum of FeGPNPs. **d**–**f** High-resolution XPS spectra of N 1s, Fe 2p, and P 2p, respectively. **g** TEM images of nanoparticles obtained using different metal ions and nucleotides

To develop sustainable and efficient nano ferroptosis inducers, GMP, a type of nucleotide, was selected as a building block with Fe^2+^ ions. Nucleotides can serve as supramolecular motifs to construct ordered architecture through coordinated interactions with metal ions [28]. Herein, GMP was first mixed with FeCl_2_, then transferred to Tris–HCl buffer solution containing dopamine. After the self-polymerization of dopamine to produce polydopamine (PDA) [29, 30], a black, turbid, and colloidal system was obtained, demonstrating formation of Fe^2+^/GMP/PDA nanoparticles (FeGPNPs) (Additional file 1: Fig. S1a). Nanostructure morphology and size were characterized by scanning electron microscopy (SEM) and transmission electron microscopy (TEM) (Additional file 1: Fig. S1b; Fig. 1a), which showed spherical and core–shell nanoparticles with a diameter of ~ 100 nm and wall thickness of ~ 25 nm. Elemental mapping using TEM energy dispersive X‐ray (EDX) spectroscopy presented clear C, N, O, P, and Fe signals on the nanoparticles (Fig. 1b), and merged images further clarified the core–shell structure. Based on X-ray photoelectron spectroscopy (XPS) patterns, C 1s (64.5 at.%), N 1s (8.2 at.%), O 1s (24.8 at.%), Fe 2p (2.0 at.%), and P 2p (0.5 at.%) peaks were identified in the FeGPNP spectrum (Fig. 1c–f). Of note, the appearance of the P signal confirmed the presence of GMP in the FeGPNPs. In addition, Fe in the obtained FeGPNPs was analyzed by a spin-coupled doublet for curve fitting of Fe 2p3/2 and Fe 2p1/2 at 711.0 and 723.7 eV, respectively, indicating that both Fe^2+^ and Fe^3+^ species existed in the FeGPNPs [31, 32]. Further analysis of XPS data showed that the weight ratio of Fe^2+^ and Fe^3+^ in total Fe was 85:15. The existence of a small amount of Fe^3+^ was attributed to the oxidation of Fe^2+^ in air. As measured by inductively coupled plasma-atomic emission spectrometry (ICP‐AES), total Fe content in FeGPNPs was 3.06 ± 0.14 wt.%. Moreover, dynamic light scattering (DLS) was utilized to determine the zeta potential and size of the FeGPNPs. Results showed a unimodal size distribution with a hydrodynamic diameter of ~ 250 nm (Additional file 1: Fig. S2a), slightly larger than the TEM and SEM results, and a zeta potential of − 10.3 mV (Additional file 1: Fig. S2b). Thus, these results indicate successful FeGPNP formation.

In addition to GMP, we found other nucleotides, including cytidine monophosphate (CMP), adenosine monophosphate (AMP), and uridine monophosphate (UMP), could form nanoparticles in the presence of Fe^2+^ ions and dopamine (Fig. 1g). We also tested whether other metal ions, including Cu^2+^, Zn^2+^, and Mn^2+^, can promote nanoparticle formation (Fig. 1g). Results showed that sphere-like nanoparticles were also formed when mixing these components. Thus, the formation of the FeGPNPs appeared to be driven by the complexation of metal ions with phosphate groups in the nucleotides [33], and different nucleotides required different metal ions to induce nanoparticle formation. Additionally, the color of the FeGPNPs/ethylene diamine tetraacetic acid (EDTA) mixture turned from black to brown, suggesting that EDTA destroyed the coordination between Fe^2+^ and GMP (Additional file 1: Fig. S3). To verify the importance of interactions between GMP and Fe^2+^, we synthesized Fe^2+^/PDA nanoparticles (FePNPs) in the absence of GMP. As seen in the TEM image of FePNPs in Fig. S4, only spherical nanoparticles (~ 110 nm) rather than core–shell structures were obtained. Thus, in the process of FeGPNPs formation, Fe^2+^ first coordinated with the phosphate groups in GMP, after which dopamine self-polymerized to form the core–shell structure.

**Fig. 2:**
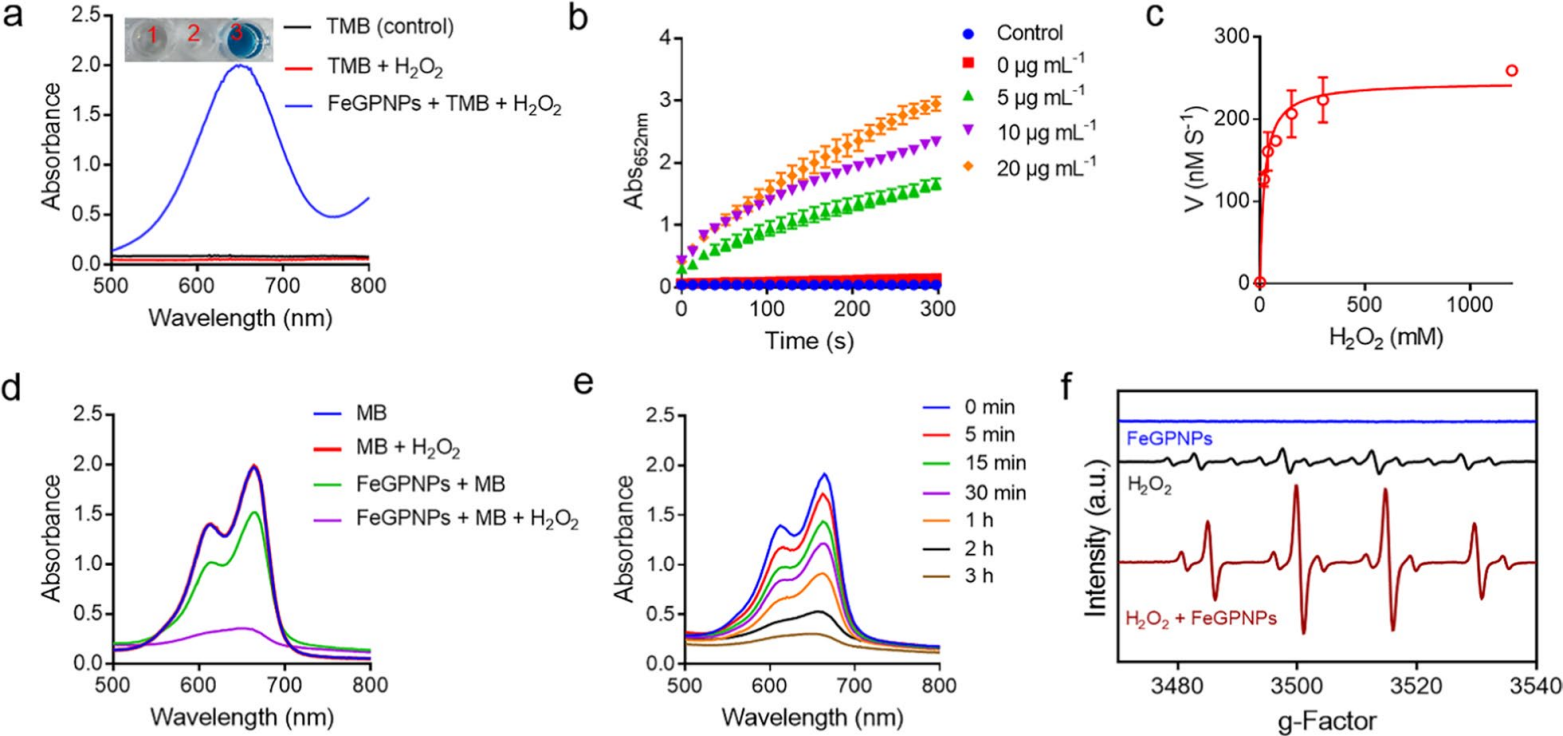
ROS generation by catalytic activities of FeGPNPs. **a** UV–vis absorbance spectra and color changes of TMB in different reaction systems after 5 min of incubation at 37 °C. 1-TMB; 2-TMB + H_2_O_2_; 3-FeGPNPs + TMB + H_2_O_2_. **b** Time-dependent absorbance changes at 652 nm using different concentrations of FeGPNPs as Fenton catalysts. **c** Kinetic assay for catalytic activity of FeGPNPs with H_2_O_2_ substrate. **d** Degradation of MB triggered by H_2_O_2_, FeGPNPs, and FeGPNPs + H_2_O_2_ after 3 h of incubation at 37 °C. **e** Time-dependent UV–vis spectra of MB degradation caused by FeGPNPs + H_2_O_2_. **f** Generation of ·OH by FeGPNPs in presence of 1.0 mM H_2_O_2_, determined by ESR

To evaluate the capacity of FeGPNPs to transform H_2_O_2_ into ·OH using the encapsulated Fe ions as Fenton catalysts, we performed the colorimetric method based on the oxidation of 3,3′,5,5′-tetramethylbenzidine (TMB) with H_2_O_2_ [34]. As shown in Additional file 1: Fig. S5, FePNPs and FeGPNPs alone could not oxidized TMB into oxidized TMB (oxTMB, blue color). However, after adding H_2_O_2_, the bright blue color could be observed. Moreover, compared with FePNPs, the presence of GMP increased the catalytic activity of FeGPNPs, consistent with the previous reports that use of nucleotides improve the peroxidase activity displayed by iron oxide and other particles [35, 36]. More specifically, FeGPNPs exhibited significant catalytic activity in a concentration- and time-dependent manner (Fig. 2a, b), and typical Michaelis–Menten curves of enzyme kinetics (Fig. 2c; Additional file 1: Fig. S6) were drawn within H_2_O_2_ and TMB concentrations. By fitting the Lineweaver–Burk equation, the enzyme kinetic parameters, e.g., maximum initial velocity (*V*_max_) and Michaelis–Menten constant (*K*_M_), were calculated (Additional file 1: Table S1). We also examined the effect of reaction medium pH and temperature on the catalytic ability of the FeGPNPs (Additional file 1: Fig. S7). The FeGPNPs showed efficient generation of ·OH under acidic conditions, with optimal activity at a pH of 4.5 and temperature of 37 °C, indicating that the FeGPNPs would possess higher activity under acidic conditions in vivo, such as in lysosomes (pH 4.5–5.0). The catalytic activity of the FeGPNPs in transforming H_2_O_2_ into ·OH was also evaluated based on methylene blue (MB) degradation [37]. MB molecule is a widely used probe to indicate the formation of ·OH. In the presence of ·OH, its color can gradually change from blue to colourless in a time-dependent manner. As shown in Fig. 2d, the FeGPNPs triggered high ROS production in the presence of H_2_O_2_, however H_2_O_2_ alone or FeGPNPs without H_2_O_2_ could not significantly degrade MB. The MB degradation process over time was shown in Fig. 2e, with MB almost completely degraded after 3 h. Furthermore, we used electron spin resonance (ESR) spectroscopy to verify ·OH generation with the aid of a widely used capture-agent for ·OH (5,5-dimethyl-1-pyrroline-*N*-oxide, DMPO) (Fig. 2f). Results showed that efficient production of ·OH (characteristic 1:2:2:1 signals in the ESR spectrum) was only detected with those FeGPNPs incubated with H_2_O_2_. Therefore, the efficient catalytic activity of the FeGPNPs in transforming H_2_O_2_ into ·OH was confirmed, thus suggesting potential for further biological applications.

**Fig. 3:**
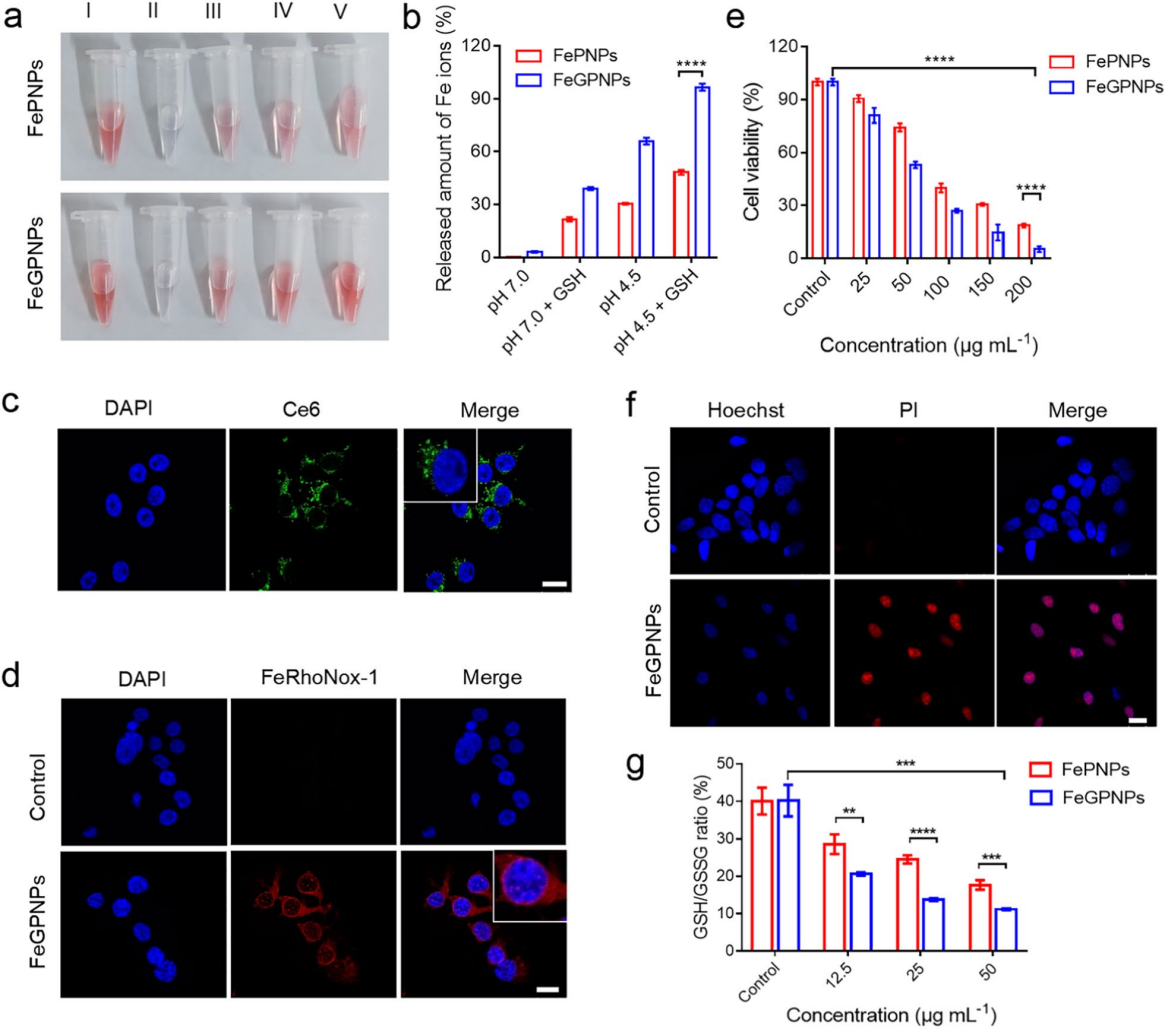
FeGPNPs released ferrous ions in CT26 cells to induce cell death. **a** Color changes in Fe^2+^ and ferrozine complexes under different conditions. I-positive control; II-pH 7.0; III-pH 7.0 + GSH 10 mM; IV-pH 4.5; V-pH 4.5 + GSH 10 mM. **b** Accumulated release of Fe ions (Fe^2+^ and Fe^3+^) at various pH (4.5 and 7.4) and GSH concentrations (0 and 10 mM). **c** CLSM images of cellular uptake of Ce6-FeGPNPs. Scale bar: 20 μm. **d** CLSM images of intracellular Fe^2+^ in CT26 cells after incubation with FeGPNPs. Scale bar: 20 μm. **e** Cytotoxicity of FeGPNPs and FePNPs against CT26 cells after 24 h of incubation. **f** CLSM images of dead cells with PI (red) staining. Hoechst-stained cell nucleus (blue). Scale bar: 20 μm. **g** GSH/GSSG ratios in FeGPNP- and FePNP-treated CT26 cells, respectively

Before evaluating the in vitro cytotoxicity of FeGPNPs, we investigated their stability under different conditions using FePNPs for comparison. Total Fe content in the FePNPs, as measured by ICP-AES, was 2.96 ± 0.12 wt.%, which was not significantly different from that in the FeGPNPs (3.06 ± 0.14 wt.%) (Additional file 1: Fig. S8). The release of Fe ions from FeGPNPs and FePNPs was monitored under different pH and glutathione (GSH) conditions using an iron colorimetric assay kit. Taking Fe^2+^ as an example, the pink Fe^2+^ and ferrozine complex darkened under low pH and high GSH concentration (10 mM), indicating that Fe^2+^ ions were released from the FeGPNPs and FePNPs (Fig. 3a). Corresponding data are demonstrated in Fig. 3b. In the absence of GSH at pH 7.0, the release of total Fe ions (Fe^2+^ and Fe^3+^) was insignificant (only 3% over 5 h), indicating that the FeGPNPs were stable in physiological environments. However, after the addition of GSH, the release of Fe ions increased markedly over the same period (up to 39%). When pH was decreased to 4.5, the release of Fe ions increased to 96%. We further identified the valency states of the released Fe ions using an iron assay kit (Additional file 1: Fig. S9). Specifically, ferrous iron (Fe^2+^) accounted for more than 88% of the total iron released by the FeGPNPs. In contrast, most ions (~ 90%) in FePNP system were ferric iron (Fe^3+^). Thus, the unique core–shell structure of the FeGPNPs prevented ferrous iron from oxidation, thereby avoiding the oxidization of highly active Fe^2+^ ions into less active Fe^3+^ ions for Fenton reaction. Importantly, compared with the FePNPs, the FeGPNPs demonstrated greater sensitivity to GSH and low pH. Additionally, the structural degradation of FeGPNPs was confirmed by TEM (Additional file 1: Fig. S10), thus demonstrating that the FeGPNPs were degraded at pH 4.5 with 10 mM GSH. Considering the tumor microenvironment (TME), with the properties of low pH and high GSH [38, 39], the FeGPNPs could be disassembled in the TME, leading to the release of the functional component (Fe^2+^ ions) of the nanoparticles at the tumor.

Cellular uptake of the FeGPNPs was also investigated using confocal laser scanning microscopy (CLSM). As seen in Fig. 3c, Ce6-labeled FeGPNPs (Ce6-FeGPNPs) entered the cells, as shown by the localization of green (Ce6) and blue (cell nuclear) fluorescence. As the main form of Fe ions in the FeGPNPs was Fe^2+^ ions, we used FeRhoNox-1, a red fluorescent probe for the detection of iron (II) [40], to detect intracellular Fe^2+^ after FeGPNPs treatment. As shown in Fig. 3d, after internalization in the tumor cells, the FeGPNPs released Fe^2+^ into the cells, resulting in a red signal increase. Importantly, intracellular oxidative stress (ROS level) was elevated after FeGPNPs treatment, as determined by 2′,7′-dichlorofluorescin diacetate (DCFH-DA) staining (Additional file 1: Fig. S11). As expected, after the FeGPNPs entered the tumor cells, they caused cellular damage via catalytic activity. As seen in Fig. 3e, after 24 h of treatment with FeGPNPs (200 μg mL^−1^), CT26 cell viability was only 5.2%, demonstrating that the FeGPNPs exhibited high cytotoxicity toward cancer cells. Notably, the FeGPNPs showed greater cytotoxicity than the FePNPs, attributed to the rapid release of more highly active Fe^2+^ ions within the TME. Additionally, typical Hoechst/propidium iodide (PI) staining confirmed that the FeGPNPs induced tumor cell death (Fig. 3f). It is worth noting that the nanoparticles obtained in the presence of other nucleotides also showed significant inhibitory effects on CT26 cells (Additional file 1: Fig. S12), indicating that nanoparticles produced via metal ion and nucleotide interactions may be universally applicable in tumor therapy. Due to the efficient catalytic activity of the FeGPNPs in ROS generation, intracellular GSH depletion may occur [41]. As shown in Fig. 3g, the glutathione/oxidized glutathione (GSH/GSSG) ratio decreased significantly in the CT26 cells incubated with FeGPNPs to a level much lower than that measured in FePNP-treated cells. Thus, the FeGPNPs, as Fenton catalysts, efficaciously killed the tumor cells in vitro by catalyzing H_2_O_2_ to produce toxic ·OH and reduce GSH. These results show that FeGPNPs can be used as effective agents for ROS generation and GSH consumption via the release of Fe ions in the TME, leading to tumor killing effects.

**Fig. 4:**
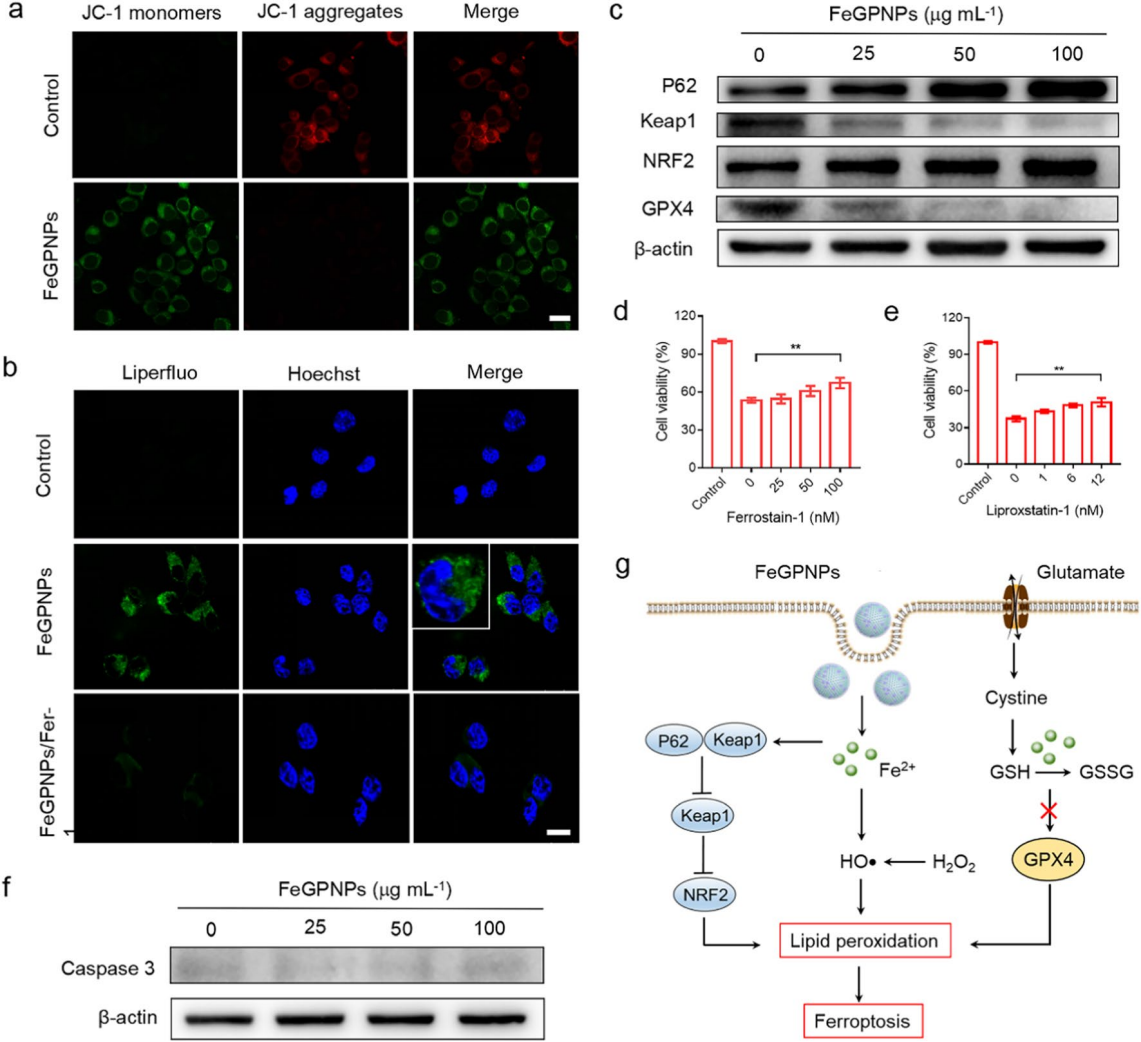
Ferroptosis caused by FeGPNPs in CT26 cells. **a** Effect of FeGPNPs on mitochondrial membrane potential. Scale bar: 20 μm. **b** CLSM images of Liperfluo staining. Scale bar: 10 µm. **c** Western blotting of GPX4, NRF2, Keap1, and P62 protein expression in FeGPNP-treated CT26 cells. **d** Effect of ferrostain-1 on cytotoxicity of FeGPNPs. **e** Effect of liproxstain-1 on cytotoxicity of FeGPNPs. **f** Cleaved caspase 3 expression levels in FeGPNP-treated CT26 cells, as determined by Western blot. **g** Schematic of FeGPNP-induced cell ferroptosis

Ferroptosis is an iron- and oxidative-dependent form of cell death induced by lipid peroxidation that induces loss of membrane integrity and subsequent cell death. To verify the occurrence of ferroptosis in tumor cells after FeGPNP treatment, various indicators of ferroptosis, including mitochondrial membrane potential, lipid peroxide, and GPX4 expression, were evaluated [8, 9]. As observed by 5,5′,6,6′-tetrachloro-1,1′,3,3′-tetraethylimidacarbocyanine (JC-1) staining, the mitochondrial membrane potential of the CT26 cells after FeGPNPs treatment declined significantly, as shown in Fig. 4a, which is an early landmark event of cell death. We next detected lipid peroxidation in CT26 cells using Liperfluo staining, which is a probe for specific indicator of lipid peroxides. As seen in Fig. 4b, the Liperfluo signal (green) increased significantly in the FeGPNP‐treated cells and the ferroptosis inhibitor ferrostain-1 (Fer-1) reduced this FeGPNP-induced effect, indicating that the FeGPNPs induced lipid peroxidation to cause ferroptosis in the FeGPNP-treated CT26 cells. GSH depletion may inactivate GPX4 in cells and hamper lipid repair systems, leading to ferroptosis [42]. As given in Fig. 4c and Additional file 1: Fig. S13, due to the GSH depletion caused by FeGPNPs (Fig. 3g), the GPX4 expression in the CT26 cells, as determined by western blotting, was markedly reduced in a dose-dependent manner after exposure to FeGPNPs. After the addition of ferroptosis inhibitors, i.e., ferrostain-1, and liproxstain-1 [43], the expression of GPX4 was partially restored (Additional file 1: Figs. S14, S15). The down-regulation of GPX4 further confirmed the occurrence of ferroptosis [44]. Similarly, ferrostain-1, liproxstain-1, and an antioxidant molecule (*N*-acetyl-l-cysteine, NAC) could inhibited the decrease in cell viability caused by the FeGPNPs, indicating that the FeGPNPs could cause tumor cell ferroptosis (Fig. 4d–e; Additional file 1: Fig. S16). To further examine the mechanisms of FeGPNP-induced tumor cell death, the expression of apoptosis-related cleaved caspase 3 in CT26 cells after FeGPNP treatment was explored. As given in Fig. 4f, there was no obvious increase in caspase 3 expression after treatment, even though the concentration of FeGPNPs reached 100 μg mL^−1^, suggesting that cell apoptosis was not initiated by the FeGPNPs.

Based on the cellular mechanisms preventing ferroptosis, nuclear factor erythroid 2-related factor 2 (NRF2) as the antioxidant transcription factor is considered to regulate the onset and outcome of ferroptosis [45], which is responsible for modulating hundreds of antioxidant genes [46]. The substrate adaptor P62 protein modulates NRF2 expression levels through direct interaction with Kelch-like ECH-associated protein 1 (Keap1) under stress [47]. As shown in Fig. 4c, following FeGPNP treatment, P62 levels in CT26 cells were elevated, which prevented NRF2 degradation and enhanced NRF2 nuclear accumulation by inactivation of Keap1. Inhibition of the P62-Keap1-NRF2 pathway rendered the CT26 cells more susceptible to ferroptosis. In addition, this phenomenon was reversed by treatment with ferrostain-1 or liproxtain-1 (Additional file 1: Figs. S14, S15). Thus, these results suggested that FeGPNPs disrupted cellular antioxidant capacity and induced ferroptosis in CT26 cells (Fig. 4g).

Before assessing the antitumor activity of the FeGPNPs in vivo, we evaluated their biosafety. Notably, the FeGPNPs could effectively cause the death of CT26 cells, but they showed no obvious cytotoxicity against the normal human liver cell line L02 (Additional file 1: Fig. S17).This tumor-specific killing effect of FeGPNPs is mostly due to the three reasons: (1) FeGPNPs showed the tumor microenvironment (TME)-specific drug release behavior. (2) The higher level of H_2_O_2_ in tumor cells induced the promoted production of ·OH after FeGPNPs treatment [48]; (3) Tumor cells show a higher demand for iron to enable rapid growth, rendering them more sensitive to iron-regulated ferroptosis [49]. Biosafety analysis in healthy mice administered 25 mg kg^−1^ of FeGPNPs was also evaluated for 7–14 days. Considering that potential risks of nanomaterials are inflammatory response, standard hematology tests were conducted. Results showed no significant changes in biochemistry and blood cell counts at 7 days after FeGPNP treatment (Additional file 1: Fig. S18a–e). In addition, the blood levels of alanine transaminase (ALT), alkaline phosphatase (ALP), aspartate transaminase (AST), creatinine (CREA), and urea in the mice after FeGPNP treatment did not differ significantly to that in the phosphate-buffered saline (PBS)-treated group at 7 days post injection (Additional file 1: Fig. S18f–j), thus demonstrating that the FeGPNPs had no marked toxicity on the kidney or liver. Similarly, the FeGPNPs showed high compatibility within the 14-day evaluation period (Additional file 1: Fig. S19). Histological evaluation via hematoxylin and eosin (H&E) staining, including heart, liver, spleen, lung, and kidney sections, also indicated that no tissue or cell damage were observed in FeGPNP-treated mice (Additional file 1: Fig. S20). Thus, the FeGPNPs are well-tolerated and biocompatible in healthy mice.

**Fig. 5:**
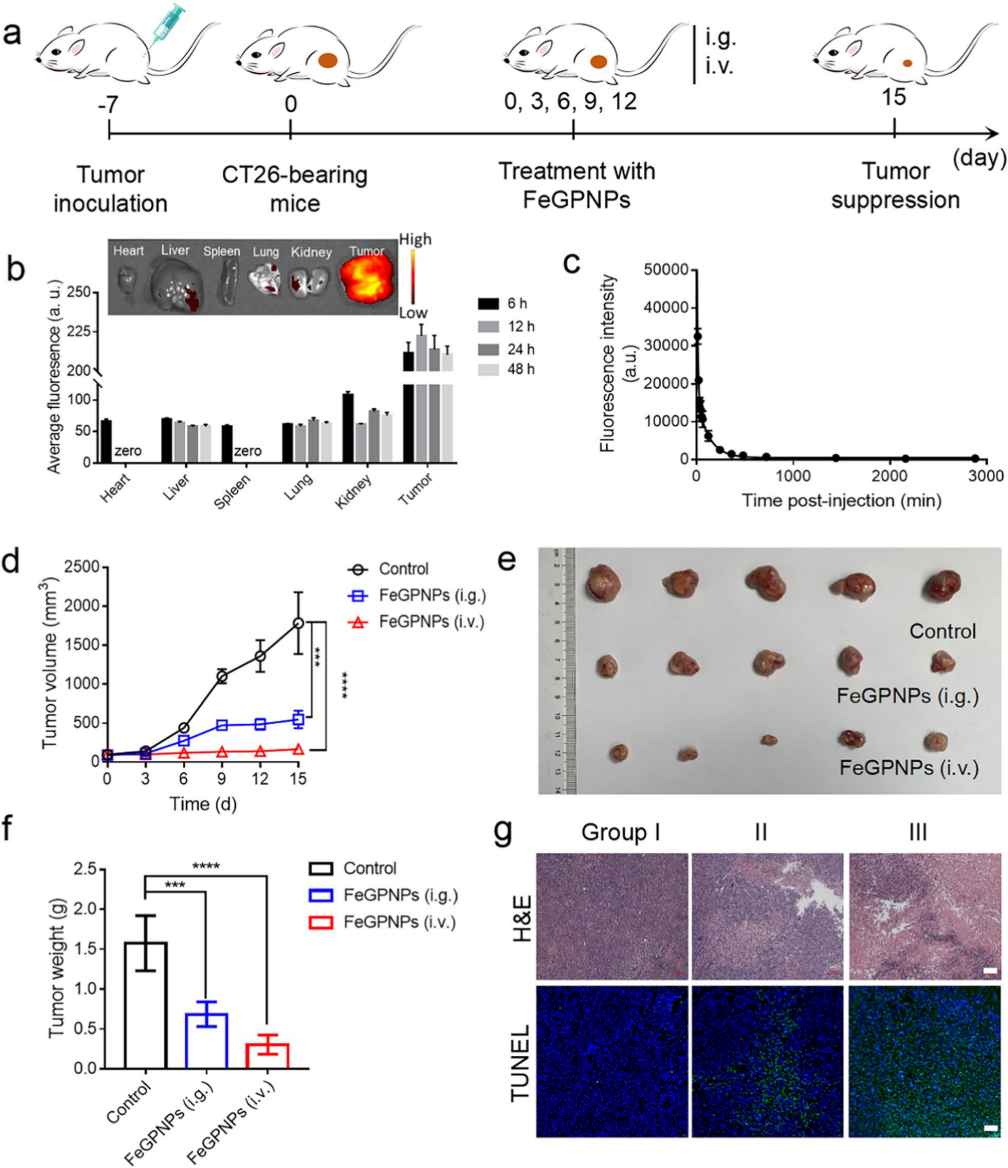
In vivo tumor therapy using FeGPNPs in CT26-xenografted mice. **a** Schematic of experiment design. **b** Semi-quantitative analysis of ex vivo fluorescence images in major organs at different time points post i.v. injection of FeGPNPs. Inset: Ex vivo fluorescence images of major organs and tumor dissected from CT26 tumor-bearing mice taken at 12 h post i.v. injection of FeGPNPs. **c** Blood circulation curve of intravenously injected Ce6 labelled FeGPNPs (Ce6- FeGPNPs). Data are means ± SD (n = 3). **d** Changes of tumor size during therapy. **e** Digital images of tumors from CT26-bearing mice after 15 days of different treatments. **f** Average tumor mass excised from CT26-bearing mice after different treatments. **g** H&E (Scale bar: 100 μm) and TUNEL (Scale bar: 50 μm) staining of tumor sections from tumor-bearing mice. Group I: PBS-treated; Group II: FeGPNP-treated (i.g.); Group III: FeGPNP-treated (i.v.)

The in vivo treatment efficacy of the FeGPNPs was assessed in mice bearing CT26 tumors (Fig. 5a). We first tracked FeGPNPs in CT26 tumor-bearing Balb/c mice in vivo through fluorescence imaging (Fig. 5b). After intravenous injection of Ce6-FeGPNPs, the Ce6 signals in the tumor site increased and peaked at 12 h post injection, showing efficient intratumor accumulation of FeGPNPs, the semi-quantitative biodistribution based on ex vivo imaging of the tumor and major organs indicated high tumor uptake of the FeGPNPs. Additionally, the blood circulating half-life of FeGPNPs was determined to be 83 min (Fig. 5c). Next, fifteen tumor-bearing mice with tumor volumes of ∼100 mm^3^ were divided into three groups (*n* = 5): Group I, tail vein injection of PBS; Group II, gavage administration (i.g.) of FeGPNPs; Group III, tail vein injection (i.v.) of FeGPNPs. On days 0, 3, 6, 9, and 12, the FeGPNPs were administered to treat the corresponding groups of mice. From day 0, tumor size and body weight were measured. Results showed that FeGPNPs treatment via gavage and tail vein injection significantly inhibited tumor growth (Fig. 5d–f). Notably, average body weights under the different treatments showed negligible variation (Additional file 1: Fig. S21), indicating excellent biosafety of the FeGPNPs. The tumor tissues were collected for H&E and terminal deoxynucleotidyl transferase dUTP nick-end labeling (TUNEL) staining (Fig. 5g). Based on H&E staining, the tumor tissues from the FeGPNP-treated mice showed many gaps in loose tissue and reduced tumor cells. The TUNEL staining results confirmed that the FeGPNPs caused efficient cell death. Additionally, histological analyses of the major organs showed that the FeGPNPs did not cause obvious pathological changes in these tissues (Additional file 1: Fig. S22). Thus, the FeGPNPs exhibited excellent ferroptosis antitumor activity in vivo.

**Fig. 6:**
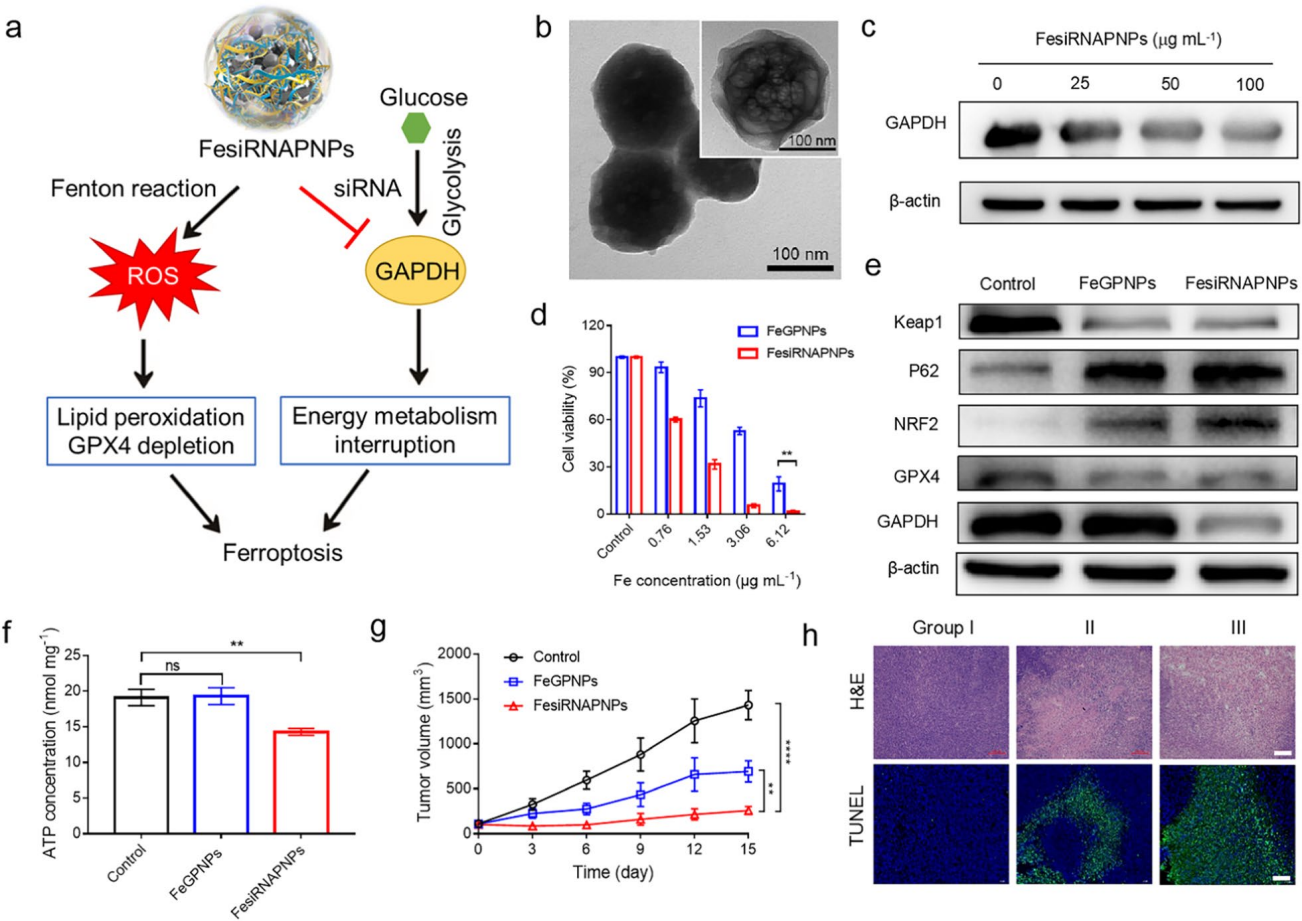
Synergistic ferroptosis/glycolysis interference by FesiRNAPNPs. **a** Schematic of FesiRNAPNPs for synergistic ferroptosis with energy metabolism interference in tumors. **b** TEM images of FesiRNAPNPs. **c** Western blot analysis of GAPDH protein levels in FesiRNAPNP-treated CT26 cells. **d** Cytotoxicity of FeGPNPs and FesiRNAPNPs against CT26 cells after 24 h of incubation. Concentration is expressed as Fe ion content. **e** Western blot analysis of GPX4, NRF2, Keap1, P62, and GAPDH protein expression levels in FesiRNAPNPs-treated CT26 cells. **f** ATP concentration in FeGPNP- and FesiRNAPNP-treated CT26 cells. **g** Tumor size change during therapy. **h** H&E (Scale bar: 100 μm) and TUNEL (Scale bar: 50 μm) staining of tumor sections in different groups. Group I: PBS-treated; Group II: FeGPNP-treated via tail vein injection; Group III: FesiRNAPNP-treated via tail vein injection

As mentioned above, nanoparticles constructed via metal ion and nucleotide interactions may be universally applicable in tumor therapy. To expand our synthesis strategy, we replaced GMP with functional nucleotide derivatives. Considering that tumor cells can activate adaptive metabolic responses to suppress ferroptosis for self-preservation, e.g., up-regulation of glycolysis, GAPDH siRNA was chosen to integrate ferroptosis and energy metabolism intervention into one vehicle. GAPDH is an important enzyme in glycolysis and commonly up-regulated in a variety of cancers [50, 51]. Therefore, targeting GAPDH to inhibit glycolysis is important for ferroptosis-based therapeutic strategies [52, 53]. Here, GAPDH siRNA was used to down-regulate GAPDH expression in cells and inhibit glucose consumption. Following the same synthesis procedure as above, hybrid nanoparticles composed of Fe ions, GAPDH siRNA, and PDA were obtained (FesiRNAPNPs) and used for synergistic ferroptosis/glucose consumption therapy in tumors (Fig. 6a). As seen in the TEM images in Fig. 6b, the FesiRNAPNPs also showed spherical morphology with a ~ 25-nm shell. We also evaluated their activity as Fenton catalysts (Additional file 1: Figs. S23, S24; Table S1), which was slightly lower than that of the FeGPNPs. Real-time polymerase chain reaction (PCR) was performed to evaluate the effect of the FesiRNAPNPs on GAPDH gene expression. As shown in Additional file 1: Fig. S25a, after FesiRNAPNPs treatment, GAPDH gene expression in the CT26 cells was remakedly reduced compared with that in the control. Western blot analysis also revealed a notable decrease in GAPDH expression after FesiRNAPNPs treatment (Fig. 6c). Moreover, we determined the siRNA loading amount to be ~ 4.0 wt.%, and FesiRNAPNPs also possessed the pH-responsive release behavior (Additional file 1: Fig. S25b). Thus, the FesiRNAPNPs showed a sequence-specific effect on the CT26 cells, i.e., they entered the cells and released specific siRNA to inhibit the expression of the target gene (GAPDH). These results indicate that FesiRNAPNPs have the potential to treat cancer by carrying GAPDH siRNA to silence target genes.

To verify the in vitro anticancer activity of FesiRNAPNPs, we performed a cell proliferation assay. As seen in Fig. 6d, the CT26 cells were incubated with different concentrations of FesiRNAPNPs and FeGPNPs, respectively. With the increase in FesiRNAPNPs concentration, toxicity to the CT26 cells increased. Importantly, the FesiRNAPNPs exhibited higher toxicity to the CT26 cells compared with that of the FeGPNPs at the same Fe ion content, showing combined antitumor effects. Simultaneously, the intracellular ROS levels and lipid peroxidation were promoted by the treatment of FesiRNAPNPs (Additional file 1: Figs. S26, S27), and the western blot results in Fig. 6e and Additional file 1: Fig. S28 indicated that the FesiRNAPNPs induced lethal ferroptosis in tumor cells and the loaded GAPDH siRNA improved GAPDH silencing efficacy. Consequently, compared with that in the control and FeGPNP groups, ATP content decreased in the CT26 cells after FesiRNAPNPs treatment, indicating that tumor cell glycolysis was successfully inhibited (Fig. 6f). Thus, these results indicate the existence of both ferroptosis and energy metabolism inhibition in the cell death mechanism caused by FesiRNAPNPs.

With encouragement of in vitro results, we next examined the in vivo performance of FesiRNAPNPs. Briefly, CT26 tumor‐bearing mice were received with an intravenous dose of FesiRNAPNPs or FeGPNPs with the same concentration of Fe ions (Additional file 1: Fig. S29a). First, we confirmed that FesiRNAPNPs showed efficient intratumor accumulation in CT26 tumor-bearing Balb/c mice, and the blood circulating half-life of FesiRNAPNPs was determined to be 98 min (Additional file 1: Fig. S29b, c). Then, the tumor size was then checked continuously for 15 days. As shown in the tumor growth curves (Fig. 6g), both FesiRNAPNPs and FeGPNPs significantly suppressed tumor growth, although the degree of tumor inhibition was higher for FesiRNAPNPs. Considering that the catalytic activity of the FesiRNAPNPs was lower than that of the FeGPNPs, the higher inhibition efficiency of the FesiRNAPNPs indicates a strong positive response to ferroptosis therapy after energy metabolism interference. Images of tumors and weights of each group were recorded (Additional file 1: Fig. S29d–e). Results showed that body weights of mice in each group did not vary greatly (Additional file 1: Fig. S30). After 15 days of treatment, all mice were sacrificed, and the tumors and major organs were harvested for histological examination. As shown in Fig. 6h, H&E and TUNEL staining of the tumor tissues confirmed that the FesiRNAPNPs had better therapeutic effects than the FeGPNPs. Therefore, the FesiRNAPNPs achieved combined antitumor effects in vivo by inducing ferroptosis and inhibiting glycolysis. Furthermore, no major abnormalities in the stained sections were observed, further confirming the minimal toxicity of the FesiRNAPNPs (Additional file 1: Fig. S31). Tumors in the FesiRNAPNP-treated mice showed more apoptotic and necrotic cells than those treated with FeGPNPs. Moreover, biosafety analyses based on cytotoxicity against L02 cells, blood analysis, and H&E staining (Additional file 1: Figs. S32–S35) showed that FesiRNAPNPs were also biocompatible and well-tolerated in healthy mice.

## Conclusions

In summary, a facile approach to synergize ferroptosis and energy metabolism interference for tumor therapy was developed by employing Fe^2+^ ion-driven assembly of nucleotides through coordinated interactions. The unique core–shell structure of the Fe/nucleotide nanoparticles protected the highly active Fe^2+^ ions from oxidization into the less active Fe^3+^ ions. Importantly, these nanoparticles were stable in physiological environments and able to release Fe^2+^ ions in the TME. These released Fe^2+^ ions triggered ferroptosis in the tumor, showing hallmarks of lipid peroxidation and GPX4 depletion. After adding siRNA to the nanoparticles, an antitumor strategy showing a strong positive response to ferroptosis therapy and energy metabolism interference was achieved. The as-prepared FesiRNAPNPs demonstrated specific suppression of GAPDH expression and thus inhibited tumor cell glycolysis, achieving significant synergy with ferroptosis to ablate tumors in vitro and in vivo. Of note, this construction strategy for antitumor therapy could be extended to other metal ions and functional nucleotides. This study proposes a new conceptual design of antitumor platform constructed from simple ligands and metal ions, thus offering a promising and versatile strategy.

## Supplementary Information

**Additional file 1**: Additional information includes SEM and TEM images of nanoparticles, size distribution and zeta potential of nanoparticles, Kinetic assay, ROS detection, cytotoxicity of nanoparticles, blood analysis, H&E staining and real time-PCR analysis.

## Data Availability

All data used to generate these results are available in the main text and supporting information.

## Abbreviations

ROS: Reactive oxygen species
siRNA: Small interfering RNA
GPX4: Glutathione peroxidase 4
GAPDH: Glyceraldehyde-3-phosphate dehydrogenase
GMP: Guanosine monophosphate
PDA: Polydopamine
·OH: Hydroxyl radicals
SEM: Scanning electron microscopy
TEM: Transmission electron microscopy
XPS: X-ray photoelectron spectroscopy
ICP‐AES: Inductively coupled plasma-atomic emission spectrometry (ICP‐AES)
DLS: Dynamic light scattering
CMP: Cytidine monophosphate
AMP: Adenosine monophosphate
UMP: Uridine monophosphate
EDTA: Ethylene diamine tetraacetic acid
MB: Methylene blue
DMPO: 5,5-Dimethyl-1-pyrroline-*N*-oxide
ESR: Electron spin resonance
TME: Tumor microenvironment
GSH: Glutathione
DCFH-DA: 2′,7′-Dichlorofluorescin diacetate
PI: Propidium iodide
JC-1: 5,5′,6,6′-Tetrachloro-1,1′,3,3′-tetraethylimidacarbocyanine
NRF2: Nuclear factor erythroid 2-related factor 2
ALT: Alanine transaminase
ALP: Alkaline phosphatase
AST: Aspartate transaminase
CREA: Creatinine
NAC: *N*-Acetyl-l-cysteine
