# Supplementary material for: Metal ions/nucleotide coordinated nanoparticles comprehensively suppress tumor by synergizing ferroptosis with energy metabolism interference
Source: J Nanobiotechnology. 2022 Apr 26;20:199. doi: 10.1186/s12951-022-01405-w (PMC9044594; doi:10.1186/s12951-022-01405-w)
Supplement: Supplementary file 1 — Additional file 1: Additional information includes SEM and TEM images of nanoparticles, size distribution and zeta potential of nanoparticles, Kinetic assay, ROS detection, cytotoxicity of nanoparticles, blood analysis, H&E staining and real time-PCR analysis. [file 12951_2022_1405_MOESM1_ESM.docx]

Supporting Information

Metal Ions/Nucleotide Coordinated Nanoparticles Comprehensively Suppress Tumor by Synergizing Ferroptosis with Energy Metabolism Interference

Yanqiu Wang^1^, Jie Chen^1^, Jianxiu Lu^1^, Juqun Xi^1,2^*, Zhilong Xu^3^, Lei Fan^3^,

Hua Dai^1,2^, Lizeng Gao^4^*

^1^ *School of Medicine, Institute of Translational Medicine, Yangzhou University, Yangzhou, 225009, PR China*

^2^ *Jiangsu Key Laboratory of Integrated Traditional Chinese and Western Medicine for Prevention and Treatment of Senile Diseases, Yangzhou, 225009, PR China*

^3^ *School of Chemistry and Chemical Engineering, Yangzhou University, Yangzhou 225002, PR China*

*^4^ CAS Engineering Laboratory for Nanozyme, Key Laboratory of Protein and Peptide Pharmaceutical Institute of Biophysics, Chinese Academy of Sciences, Beijing, 100101, PR China*

*Corresponding author*

*E-mail address: xijq@yzu.edu.cn (J. Xi), gaolizeng@ibp.ac.cn (L. Gao).*

***Materials and Methods***

***Materials.*** Phosphate buffer saline (PBS), sodium acetate (NaAc), and ethanol were purchased from Sinoparm Chemical Reagent Co., Ltd (Shanghai, China). 1-(3-Dimethylaminopropyl)-3-ethylcarbodiimide hydrochloride (EDC), 1-hydroxy-2, 5-pyrrolidinedione (NHS), ferroptosis inhibitor ferrostain-1 and liproxtain-1, and 3, 3′, 5, 5′-Tetramethylbenzidine (TMB, ≥ 99.5%) were obtained from Sigma-Aldrich (Massachusetts, USA). NH_2_-PEG-NH_2_ was purchased from Macklin Biochemical Co., Ltd (Shanghai, China). Glutathione (GSH, 99%) was purchased from Aladdin Biochemical Technology Co., Ltd. (Shanghai, China). 5, 5-Dimethyl-1-pyrroline N-oxide (DMPO, > 98%), methylene blue (MB), and dopamine (DA) were bought from Adamas Reagent Co., Ltd (Shanghai, China). Dimethylsulfoxide (DMSO), mitochondrial membrane potential assay kit with JC-1, hoechst staining kit, cell counting kit-8 (CCK-8), ATP assay kit, GSH assay kit, reactive oxygen species assay kit (2′,7′-dichloroflfluorescin diacetate, DCFH-DA), 4,6-diamidino- 2-phenylindole (DAPI), and propidium iodide (PI) were obtained from Beyotime Biotechnology Co., Ltd (Shanghai, China). GAPDH siRNA was obtained from Jtsbio Biotechnology Co., Ltd (Wuhan, China). GAPDH siRNA: UGACCUCAACUACAUGGUUTT AACCAUGUAGUUGAGGUCATT. PrimeScriptTMRT reagent Kit with gDNA Eraser, custom oligo DNA were obtained from TaKaRa (Japan) (muse Actb-F 5’-CATCCGTAAAGACCTCTATGCCAAC-3’ and Actb-R 5’-ATGGAGCCACCGATCCACA-3’, mouse Gapdh-F 5’-TGTGTCCGTCGTGGATCTGA-3’ and Gapdh-R 5’-TTGCTGTTGAAGTCGCAGGAG-3’).

***Synthesis of FeGPNPs.*** FeCl_2_ and GMP were first mixed at a molar ratio of 1:2 (final concentration: 1 mM : 2 mM) and added to Tris-HCl buffer solution (50 mL, pH 8.8, 0.01 M). Then, a certain amount of DA (final concentration: 0.3 mg mL^-1^) was slowly added into the above mixture. After stirring for 6 h, the reaction was terminated. The products were washed with deionized water until the filtrate was colorless, and then were freeze-dried for further use.

***Synthesis of FePNPs.*** FeCl_2_ with the final concentration (1 mM) was first dissolved in Tris-HCl buffer solution (pH 8.8, 0.01 M, 50 mL). Then, a certain amount of DA (final concentration: 0.3 mg mL^-1^) was slowly added and stirred for 6 h. After the reaction was terminated, the products were collected through washing and freeze-dried.

***Synthesis of FesiRNAPNPs.*** FeCl_2_ and GAPDH siRNA were first mixed at the final concentrations of 1mM and 100 nM, respectively, and added to Tris-HCl buffer solution (30 mL, pH 8.8, 0.01 M). Then a certain amount of DA (final concentration: 0.3 mg mL^-1^) was slowly added and stirred for 6 h. After the reaction was terminated, the products were collected through washing and freeze-dried.

***Characterization*.** The morphologies of the products were characterized by transmission electron microscope (TEM) (Tecnai 12, Philips, Holland; HT7800, Hitachi, Japan) and scanning electron microscope (SEM) (S4800, Hitachi, Japan). The chemical state and composition of the products were characterized by X-ray photoelectron spectroscopy (XPS) (ESCALAB250Xi, ThermoFisher, USA). X-ray diffraction spectrum (XRD) was obtained by a D8Advance X-ray diffractometer (Bruker, Germany). Electron spin resonance (ESR) measurements were carried out using an ESR spectrometer (A300-10/12, Bruker, Germany). The content of Fe in FeGPNPs was determined by inductively coupled plasma atomic emission spectrometer (ICP-AES) (Optima 7300DV, PerkinElmer, USA). The hydrodynamic diameter and Zeta potential of FeGPNPs were measured by dynamic light scattering (DLS) (Malvern Zetasizer Nano ZS, UK). Confocal laser scanning microscopy (CLSM, Gemini SEM 300, Carl Zeiss, Germany) was adopted to observe the signals of fluorescence probes.

***Catalytic activity detection*.** The catalytic activity of FeGPNPs/FesiRNAPNPs as Fenton catalysts was first assessed using TMB as the substrate in the presence of H_2_O_2_ at 37ºC. To determine whether FeGPNPs/FesiRNAPNPs show catalytic-like activity, different concentrations (final concentration: 0, 5, 10, 20 μg mL^-1^) of FeGPNPs/FesiRNAPNPs was added into 0.1 M HAc-NaAc buffer solution (pH 4.5, total volume: 200 μL) containing TMB (final concentration: 0.832 mM) and H_2_O_2_ (300 mM). The UV-vis absorbance spectra of oxidized TMB were recorded via a microplate reader. pH dependence assay of the catalytic activity was carried out by employing 25 μg mL^-1^ FeGPNPs/FesiRNAPNPs in different pH buffers (1.0, 2.0, 3.0, 4.0, 4.5, 5.0, 6.0, 7.0, 8.0, 9.0, 10.0 11.0). The steady-state kinetic assays of FeGPNPs/FesiRNAPNPs with H_2_O_2_ as the substrate were performed by adding nanoparticles (10 μg mL^-1^) into 0.1 M HAc-NaAc buffer solution (pH 4.5, total volume: 200 μL) containing TMB (final concentration: 0.832 mM) and different concentrations of H_2_O_2_ (0, 18.75, 37.5, 75, 150, 300 and 1200 mM). The steady-state kinetic assays of FeGPNPs/FesiRNAPNPs with TMB as the substrate were performed by adding nanoparticles (10 μg mL^-1^) into 0.1 M HAc-NaAc buffer solution (pH 4.5, total volume: 200 μL) containing H_2_O_2_ (final concentration: 300 mM) and different concentrations of TMB (0, 0.078, 0.156, 0.312, 0.624, 0.832, 1.04, 1.248, 1.872 mM).

In order to detect the production of ·OH, a classical colorimetric method based on the degradation of MB in oxidative environment was used. In brief, FeGPNPs (50 μg mL^-1^) were added into an aqueous solution containing H_2_O_2_ (0.01M) and MB (15 μg mL^-1^). After incubation at 37 °C for various time intervals, the absorption of above solutions at 665 nm was measured to record the degradation of MB. In addition, ESR spectroscopy was used to evaluate the •OH production via a commonly used a radical trap agent (DMPO).

***Cell viability assay*.** The cytotoxicity of nanoparticles (FePNPs, FeGPNPs, and FesiRNANPs) was detected by a cell counting Kit-8 assay (CCK-8). Briefly, mouse colon cancer cells (CT26) were plated at 5000 cells per well in 96-well plates and allowed to settle overnight for adherence. Different concentrations of nanoparticles were then added into wells for a 24 h incubation. Then the cell viability was determined using CCK-8 according to the manufacturer’s instructions, and finally the absorbance of 450 nm was measured by a microplate reader.

As for the exploration of the inhibition effect of different inhibitors on the cytotoxicity of nanoparticles, ferroptosis inhibitor (ferrostatin-1 or liproxtain-1) were added into the cells along with nanoparticles. The inclusion time and subsequent assay were the same as described above.

***Cellular ROS assay.*** The intracellular ROS generation by FeGPNPs was measured by a fluorescent microscopy using a DCFH-DA probe. Briefly, CT26 cells were plated at 1 × 10^5^ cells per well in six well plates and allowed to settle overnight for adherence. FeGPNPs (50 μg mL^-1^) were then added into wells for a 6 h incubation. Then, the medium was removed, and cells were detached and rinsed with PBS buffer three times. The fluorescence probe DCFH-DA was added into the cells and incubated for 30 min at 37 °C in the dark. The fluorescence images of the treated cells were examined with excitation at 488 nm and emission at 530 nm and analyzed by Image J software.

***Mitochondria potential assay.*** Loss of mitochondrial membrane potential was assessed by CLSM using the dye JC-1. Briefly, CT26 cells (2 × 10^4^ cells per well) were seeded in 24-well plates for 12 h before using. After treatment with 50 μg mL^-1^ FeGPNPs for 6 h, CT26 cells were stained with JC-1 for 20 min at 37 °C. After washed twice following the manufacturer’s instructions, cells observed through CLSM analysis. Red emission of the dye represents a potential-dependent aggregation in the mitochondria. Green fluorescence represents the monomeric form of JC-1, appearing in the cytosol after depolarization of the mitochondrial membrane. Cells treated with CCCP (carbonyl cyanide 3-chlorophenylhydrazone) were used as a positive control.

***Hoechst/propidium iodide (PI) staining.*** CT26 cells were seeded in 24-well plates at a density of 2 × 10^4^ cells per well and allowed to settle overnight for adherence. FeGPNPs (50 μg mL^-1^) were then added into wells for 6 h incubation. Then, the medium was removed, and cells were detached and rinsed with PBS buffer three times. The fresh medium containing Hoechst (5 µg mL^-1^) and PI (5 µg mL^-1^) were added. After cultured for 20 min, the cells were washed with PBS and a CLSM unit was utilized to acquire fluorescence images of the cells.

***CLSM observations of cellular uptake behavior.*** FeGPNPs were first labeled by Chlorin e6 (Ce6) to prepare Ce6-FeGPNPs. CT26 (2 × 10^4^ cells per well) were seeded in a CLSM-specific dish for CLSM analysis. When the density reached ~ 80%, Ce6-FeGPNPs were added, and the cells were cultured at 37 °C in a humidified incubator for another 12 h. Then, the cells were washed with PBS three times, and the nuclei were stained with DAPI. A CLSM unit was utilized to obtain the fluorescence images of the cells.

***Fe^2+^ fluorescence staining in cells*.** CT26 cells were seeded in 24-well plates at a density of (2 × 10^4^ cells per well and grown in 5% CO_2_ at 37 ℃ overnight. After treatment with 50 μg mL^-1^ FeGPNPs for 6 h, the cells were washed by PBS and stained by Hoechst (5 µg mL^-1^) for 20 min and FeRhoNox-1(5 µM) for 1 h at 37 °C. Finally, the cells were washed with PBS twice and imaged by a laser confocal microscope to observe the fluorescence signal intensity in the cells.

***Intracellular lipid peroxide measurement.*** The intracellular lipid peroxide content was determined by CLSM. CT26 cells were seeded in 24-well plates at a density of 2 × 10^4^ cells per well and grown in 5% CO_2_ at 37 ℃ overnight. FeGPNPs (50 μg mL^-1^) were added into wells for a 6 h incubation, then the cells were stained by Hoechst (5 µM) for 20 min and Liperfluo (5 µM) for 30 min at 37 °C. A CLSM unit was used to obtain the fluorescence images of cells. The effect of the ferroptosis inhibitor (ferrostatin-1) on the intracellular lipid peroxidation were also evaluated.

***Detection of intracellular ATP content.*** CT26 cells were seeded in 6-well plates at a density of 1×10^5^ cells per well and grown in 5% CO_2_ at 37 ℃ overnight. FeGPNPs (21.6 μg mL^-1^) and FesiRNANPs (50 μg mL^-1^) with the same iron concentration were added. After incubation for 12 h, the cells collected by centrifugation in each well were mixed with 200 μL lysate, and then the supernatant was collected by centrifugation. The content of ATP in the cells was measured according to the steps of the enhanced ATP detection kit. The assay was carried out according to the manufacturer’s instructions.

***Detection of glutathione (GSH) content in the cells.*** CT26 cells were seeded in 6-well plates at a density of 1 ×10^5^ cells per well and grown in 5% CO_2_ at 37 ℃ overnight. Different concentrations of FeGPNPs and FePNPs (12.5, 25, 50 μg mL^-1^) were added into dishes for 12 h of incubation. The GSH and GSSG content was measured by employing a commercial colorimetric GSH assay kit. The assay was carried out according to the manufacturer’s instructions.

***Accumulated release profiles of Fe elements.*** Using medium with different pH values (7.4 and 4.5) and GSH concentrations (0 and 10 mM), FeGPNPs/FePNPs were added and incubated at 37 °C in dialysis tube for 5 h incubation. The concentration of Fe was measured according to the trace serum iron concentration determination kit (Solarbio, China) according to the manufacturer’s instructions.

***qPCR.*** CT26 cells were seeded in 6-well plates at a density of 1 × 10^5^ cells per well and grown in 5% CO_2_ at 37 ℃ overnight. Different concentrations of FesiRNAPNPs (25, 50, 100 μg mL^-1^) were added into the wells for 24 h of incubation. Total RNA was extracted from cells using Trizol reagent (Ambion, Carlsbad, CA, USA), and then was reverse transcribed to cDNA using PrimeScriptTMRT reagent Kit with gDNA Eraser (TaKaRa, Japan). qRT-PCR analysis was performed on a StepOnePlus Real-Time PCR system. The relative expression levels of GAPDH small nuclear RNA were normalized to the levels of β-actin small nuclear RNA.

***Western-blot.*** The expressions of GPX4, GAPDH, Keap1, P62 and NRF2 were assessed by Western blotting. Briefly, CT26 cells were inoculated on a 6-well culture plate and cultured at 37 °C for 12 h. Different concentrations of FeGPNPs/FesiRNAPNPs (0, 25, 50, 100 μg mL^-1^) were added into dishes for 12 h incubation. The cell lysates were collected, run on SDS-polyacrylamide gels, and transferred onto nitrocellulose membranes. The protein expression was analyzed using protein-specific antibodies and HRP-conjugated anti-rabbit IgG whole antibody, then visualized using a luminol-based enhanced chemiluminescence HRP substrate by fluorescence chemiluminescence analysis system, and the grayscale analysis was carried out with Image J software.

***In vitro biosafety assay of FeGPNPs and FesiRNAPNPs.*** A CCK-8 cell proliferation assay was used to evaluate cell viability. Human hepatocytes (L02) cells were seeded in a 96-well plate (approximately 5000 per well) and cultured at 5% CO_2_ and 37 °C overnight. FeGPNPs or FesiRNAPNPs were added at different concentrations and cultured for 24 h. The medium was removed, the cells were washed with PBS three times. After that, 10 μL CCK-8 solution was added to each well and incubated at 37 ℃ for 2 hours, then 80 μL supernatant was absorbed into a new 96-well plate. A microplate reader was used to measure the absorbance at 450 nm.

***In vivo*** ***biosafety assay of FeGPNPs and FesiRNAPNPs.*** All animal experiments were carried out in accordance with the guidelines approved by the Institutional Animal Care and Use Committee of Yangzhou University. For the biosafety assay of FeGPNPs, 18 healthy mice were randomly divided into 6 groups (3 mice per group), including PBS (7 days postinjection), FeGPNPs (i.v. 25 mg kg^-1^, 7 days postinjection), FeGPNPs (i.g. 25 mg kg^-1^, 7 days postinjection), FeGPNPs (i.g. 25 mg kg^-1^, 14 days postinjection), FeGPNPs (i.g. 25 mg kg^-1^, 14 days postinjection), At the indicated time, the mice were sacrificed and their blood samples were obtained to perform the standard hematology test and serum biochemistry assay. Major organs including heart, liver, spleen, lung, and kidney were fixed in 4% paraformaldehyde, embedded in paraffin, sectioned into ∼5 μm, and stained with H&E to check the state of the major organs. The evaluation of FesiRNAPNPs was conducted as the above experimental methods.

***In vivo antitumor activity of FeGPNPs and FesiRNAPNPs.*** 4 weeks old Balb/c male mice (18-22 g) were selected to establish tumor models. Briefly, 100 μL of serum-free DMEM medium suspension containing 1 × 10^6^ CT26 cells was injected subcutaneously into the right hind limb of the mouse. About 7 days later, when the tumor volume reaches about 60-100 mm^3^, the mice will used for antitumor detection.

The mice were randomly divided into 3 groups (n = 5 in each group): (1) PBS group, (2) FeGPNPs (i.g.) group, (3) FeGPNPs (i.v.) group. On the 0th, 3rd, 6th, 9th and 12th days, each group of mice was administered. The dosage under different administration modes was 25 mg kg^-1^. The tumor volume and body weight of each mouse were recorded every 3 days. The tumor volume of each mouse was calculated by the following formula: V=L×W^2^/2. Where L and W represent the maximum and minimum diameter of the tumor, respectively. On the 15th day after injection, all mice were sacrificed, and the main organs (heart, liver, spleen, lung, and kidney) were removed and stored in a 4% paraformaldehyde solution. The sample was fixed by paraffin and the section thickness was 3 μm. H&E staining and TUNEL staining were analyzed with a fluorescence microscope.

Another group of animal experiments established the subcutaneous tumor model of Balb/c mice according to the above method. The mice were randomly divided into 3 groups (n = 5 in each group): (1) PBS group, (2) FeGPNPs group, (3) FesiRNAPNPs group. All the drugs were administered by tail vain injection. On the 0th, 3rd, 6th, 9th and 12th days, each group of mice was administered. The dosage of FesiRNAPNPs group was 25 mg kg^-1^. In the case of the same concentration of Fe, the dosage of FeGPNPs group was 10.78 mg kg^-1^.


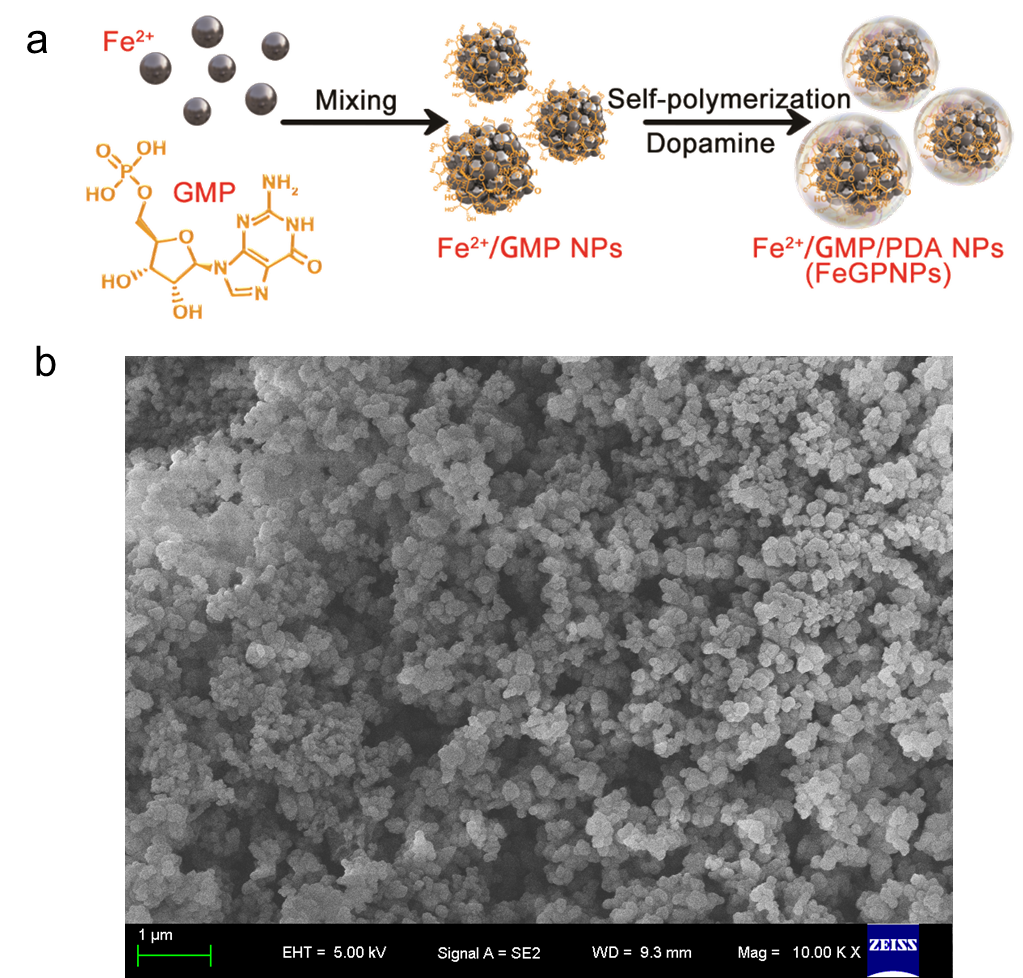


**Fig. S1.** (a) Schematic of FeGPNPs synthesis. (b) SEM images of FeGPNPs.


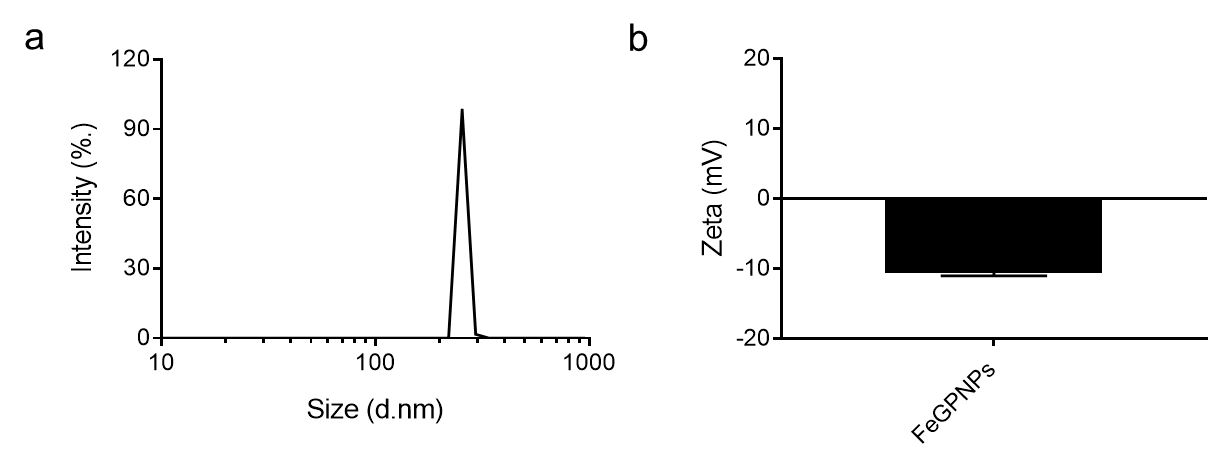


**Fig. S2.** (a) Size distribution and (b) zeta potential of FeGPNPs.


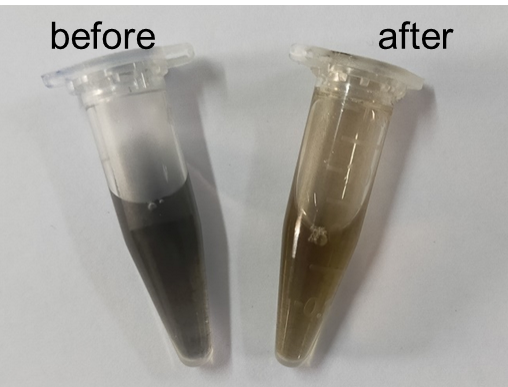


**Fig. S3.** Color change of FeGPNPs before (left) and after (right) reaction with EDTA for 6 h.


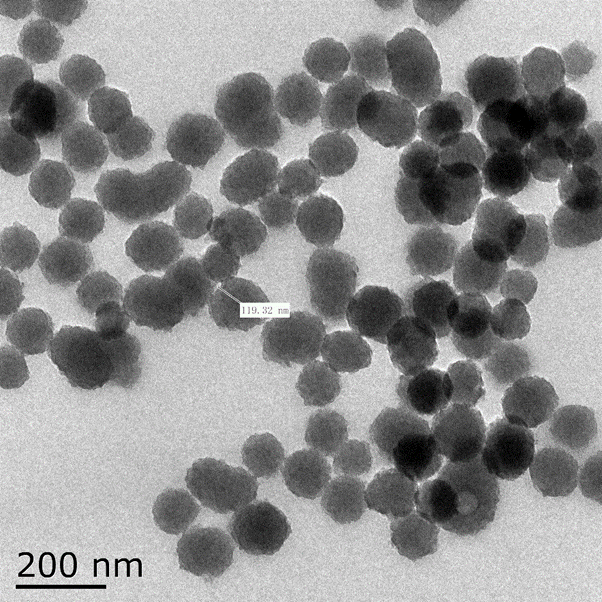


**Fig. S4.** TEM image of nanoparticles formed in the presence of Fe^2+^ ions and dopamine.


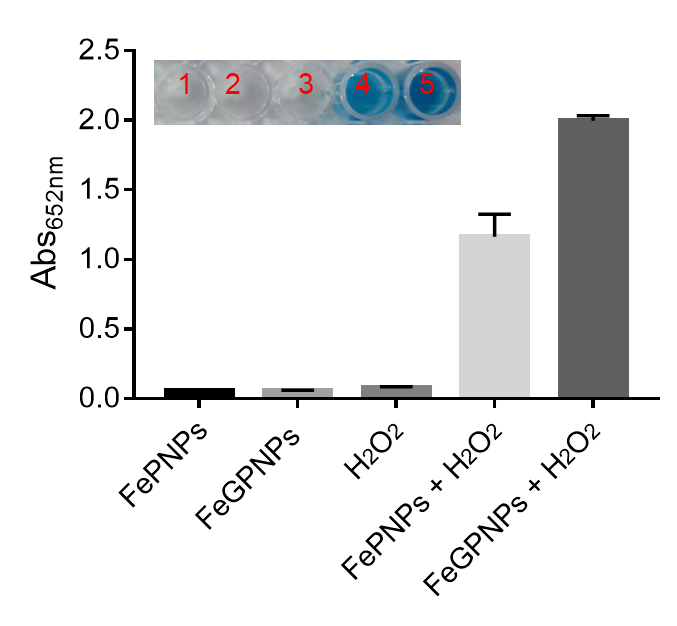


**Fig. S5.** The absorbance values at 652 nm and color changes (inset) of TMB in different reaction systems after 5 min of incubation at 37ºC. 1- FePNPs + TMB; 2-FeGPNPs + TMB; 3-H_2_O_2_ + TMB; 4-FePNPs + H_2_O_2_ + TMB; 5-FeGPNPs + H_2_O_2_ + TMB. The concentration of nanoparticles was 10 μg mL^-1^.

**Fig. S6.** Kinetic assay for the catalytic activity of FeGPNPs with TMB as substrate.


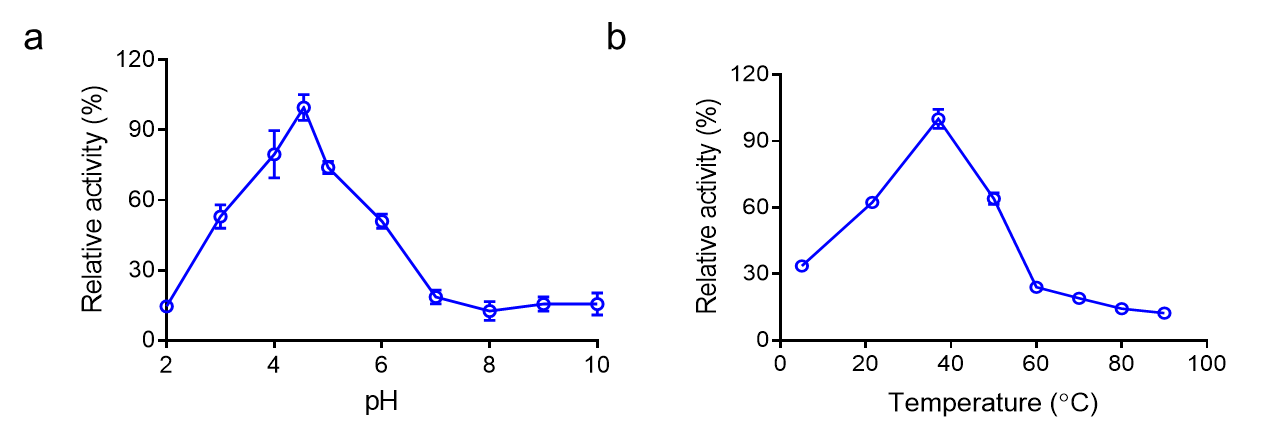


**Fig. S7.** (a) pH-dependent and (b) temperature-dependent catalytic activity of FeGPNPs. [TMB] = 0.832 mM, [H_2_O_2_] = 300 mM; [FeGPNPs] = 25 μg mL^-1^.

**Fig. S8.** Total Fe contents in FeGPNPs and FePNPs, respectively, determined by ICP-AES.

**Fig. S9**. Identification valency states of the released-Fe ions from FeGPNPs and FePNPs.


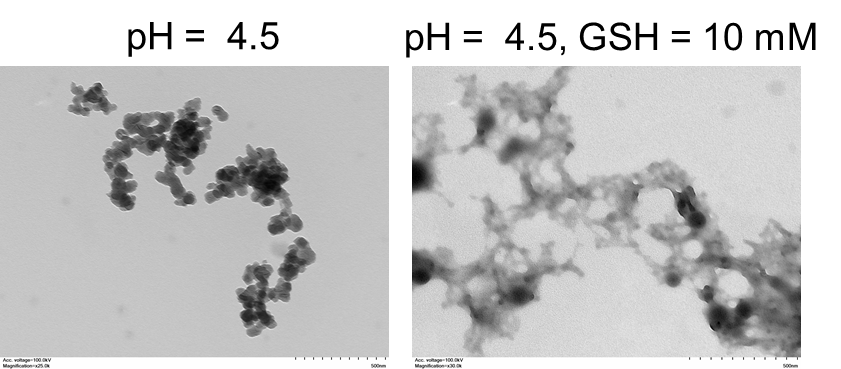


**Fig. S10.** TEM images of FeGPNPs with treatment of weak acid and GSH.


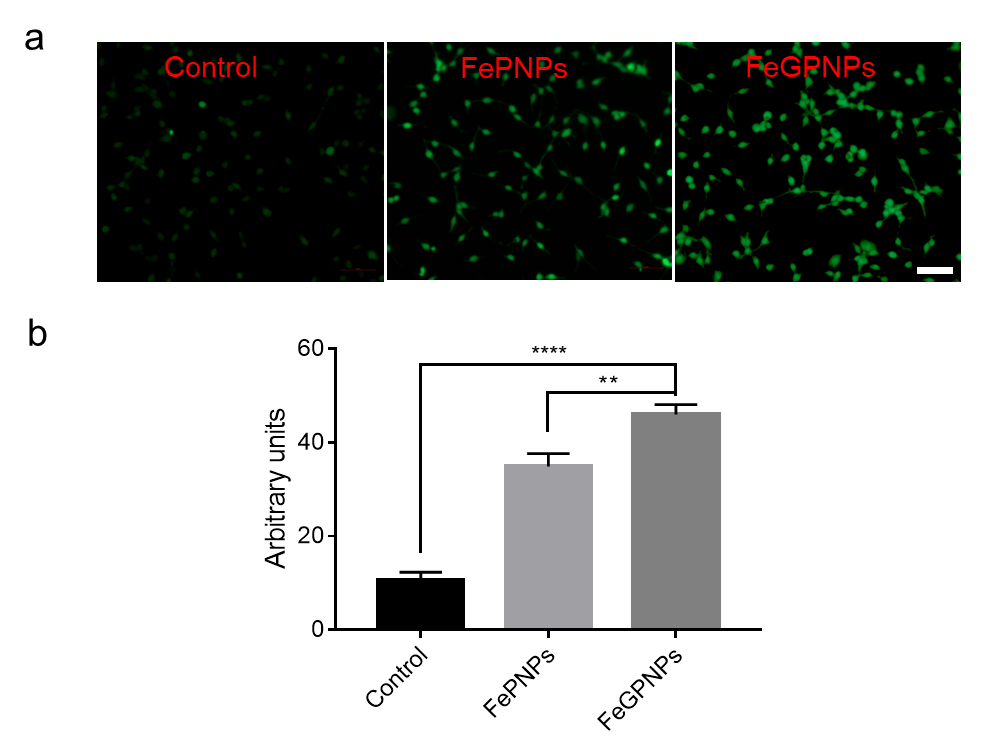


**Fig. S11.** (a) DCFH-DA assay of CT26 cells treated with FePNPs (50 μg mL^-1^) and FeGPNPs (50 μg mL^-1^). Scale bar: 50 μm. (b) Corresponding ROS level in CT26 cells after different exposure.


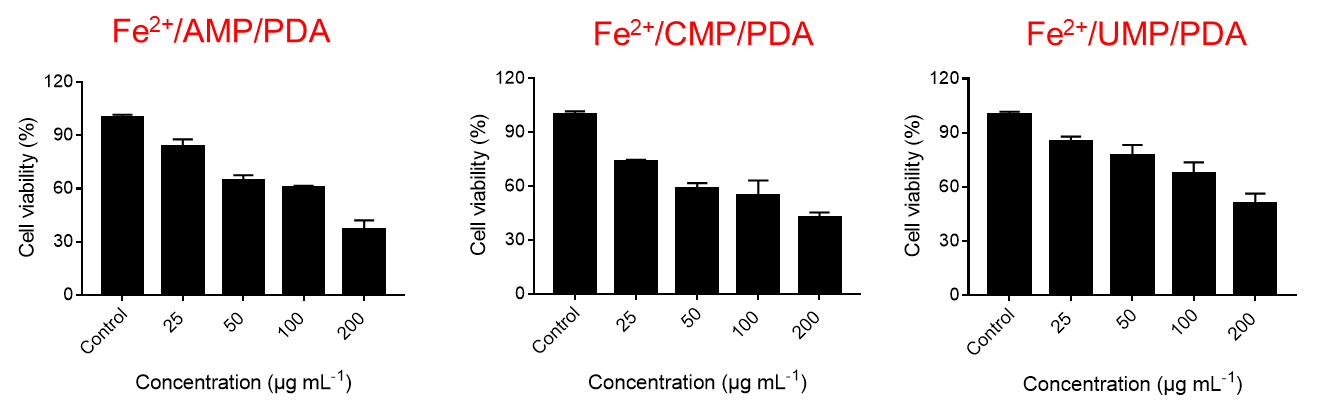


**Fig. S12.** Cytotoxicity of nanoparticles formed between Fe^2+^ and different nucleotides in the presence of dopamine against CT26 cells after 24 h of incubation.

**Fig. S13.** Quantitative analysis for GPX4, NRF2, Keap1, and P62 protein expression level in FeGPNP-treated CT26 cells.


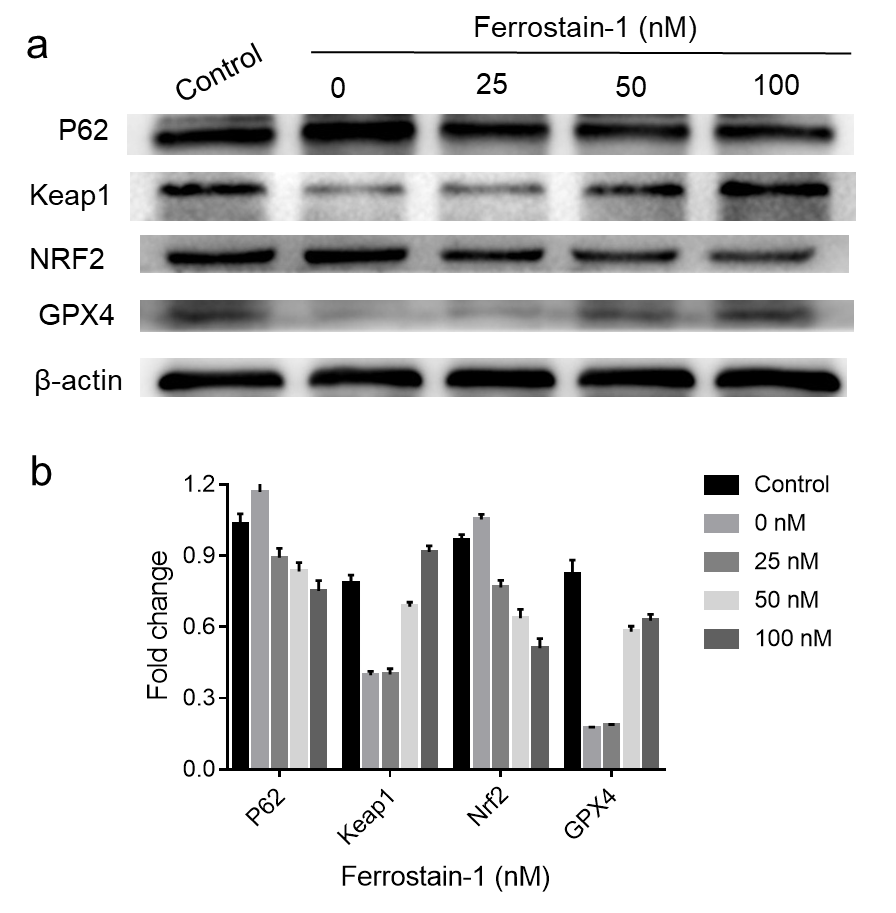


**Fig. S14.** Effect of ferroptosis inhibitor ferrostain-1 (Fer-1) on the GPX4, NRF2, Keap1, and P62 protein expression level in FeGPNP-treated CT26 cells.


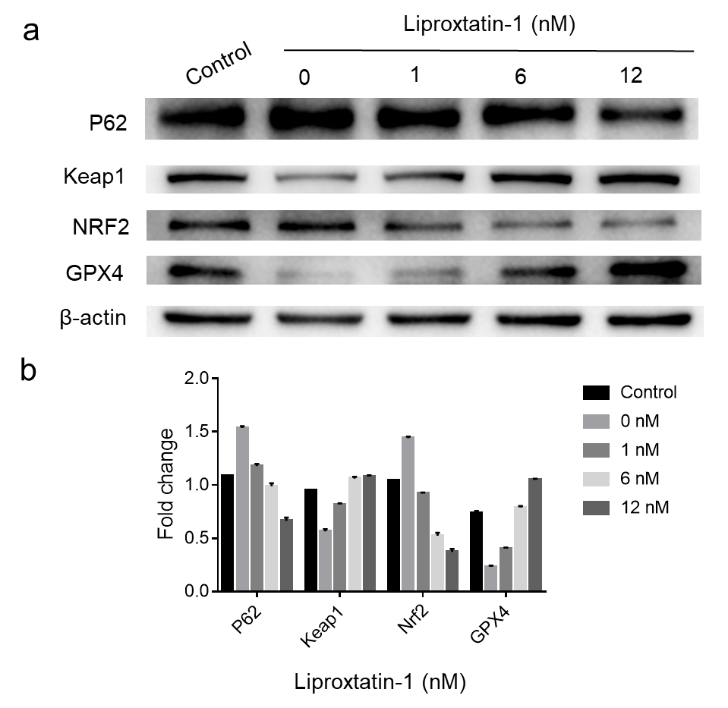


**Fig. S15.** Effect of ferroptosis inhibitor liproxstain-1 on the GPX4, NRF2, Keap1, and P62 protein expression level in FeGPNP-treated CT26 cells.

**Fig. S16.** Effect of the antioxidant molecule, NAC, on the cytotoxicity of CT26 cells treated with FeGPNPs.

**Fig. S17.** Cytotoxicity of FeGPNPs on normal cell line L02 for 24 h incubation.


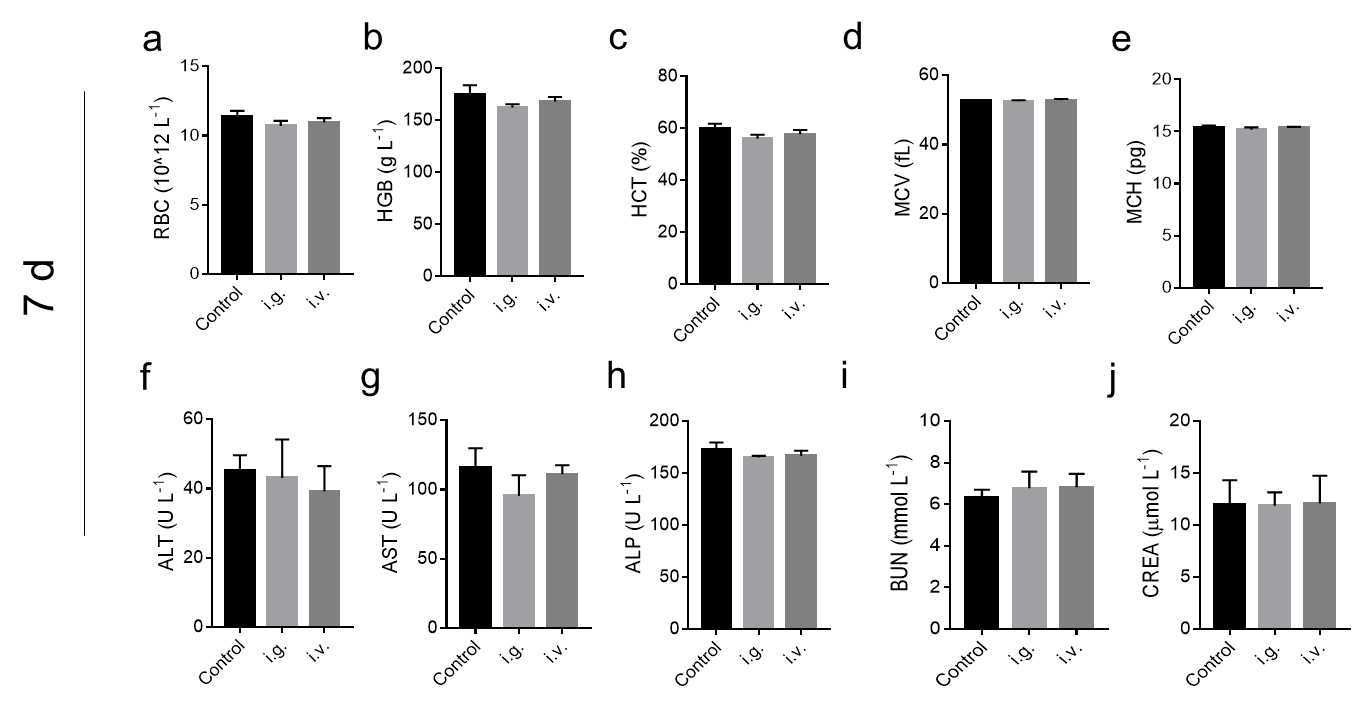


**Fig. S18.** (a-e) Blood routine data obtained through i.g. and i.v. treatment with FeGPNPs (25 mg kg^-1^) on day 7, including RBC, HGB, HCT, MCV, and MCH. (f-j) Serum biochemistry data obtained through i.g. and i.v. treatment with FeGPNPs (25 mg kg^-1^) on day 7, including liver function (ALP, ALT, and AST) and kidney function (CREA and BUN). Data are means ± SD (*n* = 3).


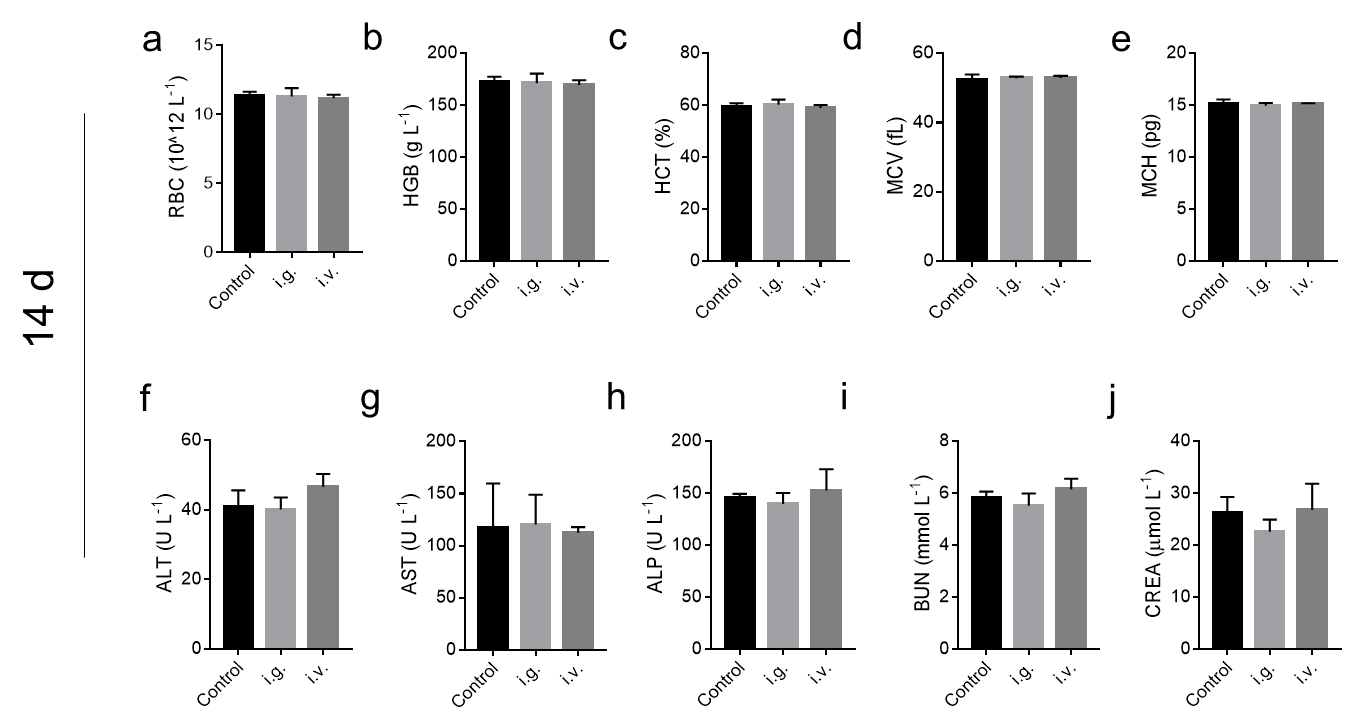


**Fig. S19.** (a-e) Blood routine data obtained through i.g. and i.v. treatment with FeGPNPs (25 mg kg^-1^) on day 14, including RBC, HGB, HCT, MCV, and MCH. (f-j) Serum biochemistry data obtained through i.g. and i.v. treatment with FeGPNPs (25 mg kg^-1^) on day 14, including liver function (ALP, ALT, and AST) and kidney function (CREA and BUN). Data are means ± SD (*n* = 3).


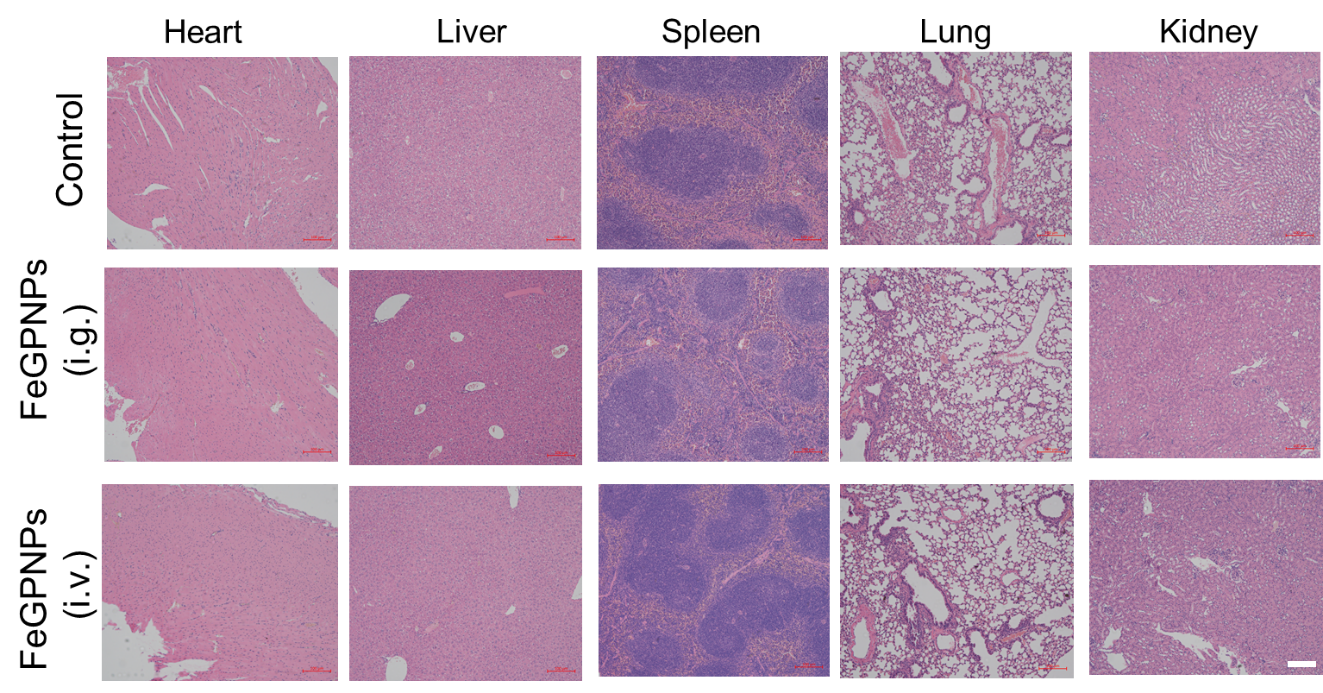


**Fig. S20.** H&E staining of the major organs (liver, spleen, heart, lung, and kidney) of healthy mice with i.g. and i.v. injection of FeGPNPs (25 mg kg^-1^). Scale bar = 100 µm.

**Fig. S21.** Body weight changes in tumor-bearing after different treatments. Data are shown as means ± SD (*n* = 5).


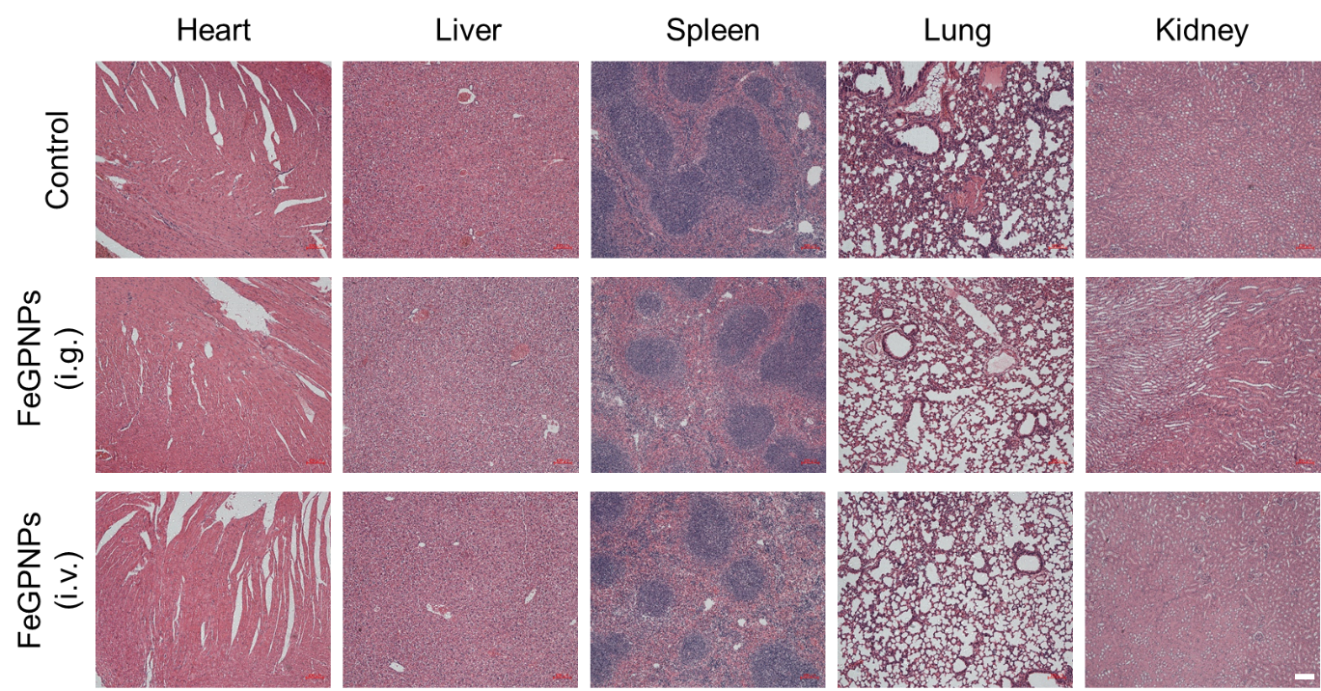


**Fig. S22.** H&E staining of the major organs (liver, spleen, heart, lung, and kidney) of tumor-bearing mice with i.g. and i.v. injection of FeGPNPs. Scale bar = 100 µm.


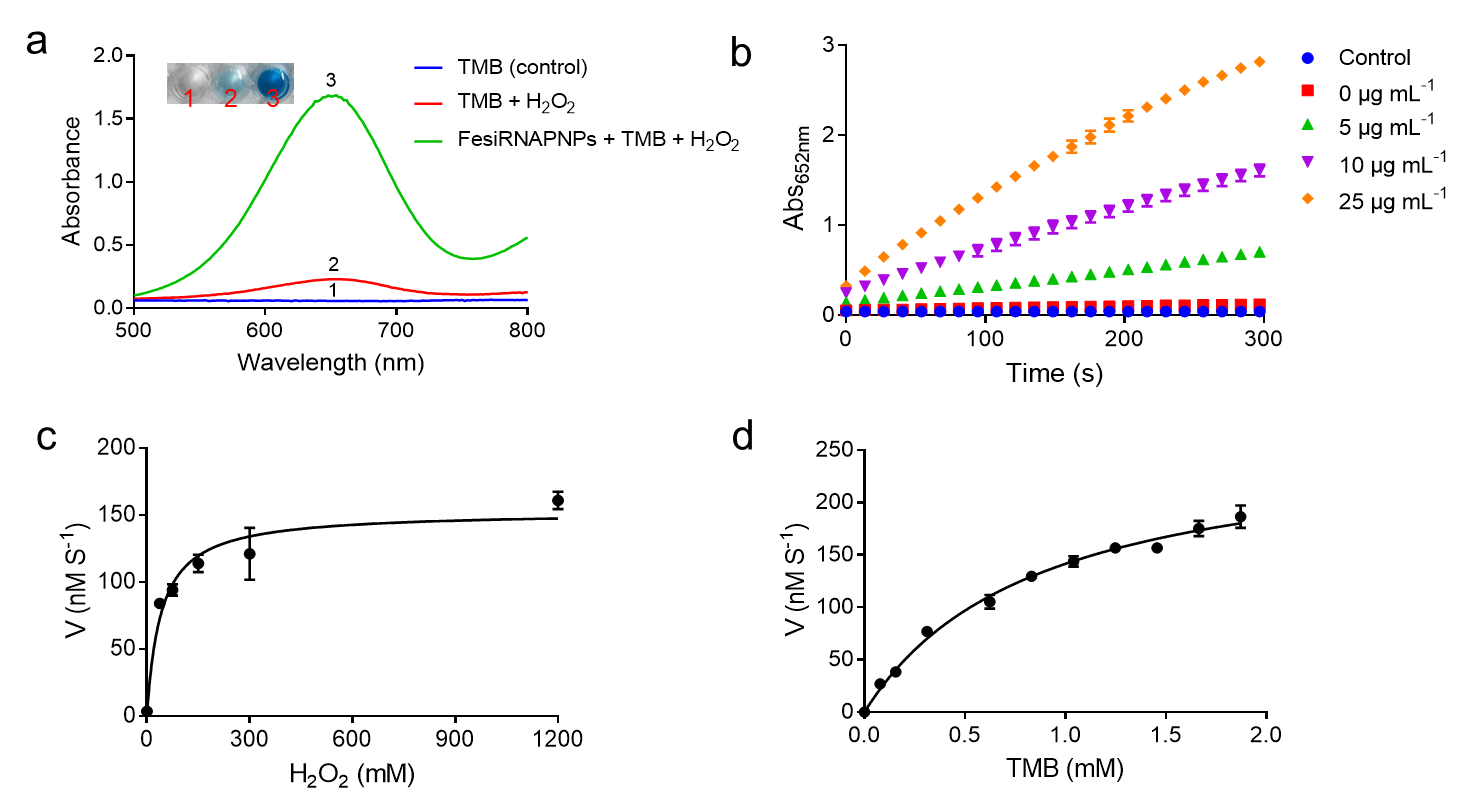


**Fig. S23.** (a) UV-vis absorbance spectra and color changes of TMB in different reaction systems: (1) Control (TMB), (2) TMB + H_2_O_2_, and (3) TMB + FesiRNAPNPs + H_2_O_2_ in pH 4.5 NaAc-HAc buffer after 5 min of incubation. The concentration of FesiRNAPNPs was 10 μg mL^-1^. (b) Time-dependent absorbance changes at 652 nm by using different concentrations of FesiRNAPNPs. (c, d) Kinetic assay for the catalytic activity of FesiRNAPNPs with H_2_O_2_ and TMB as substrates, respectively.


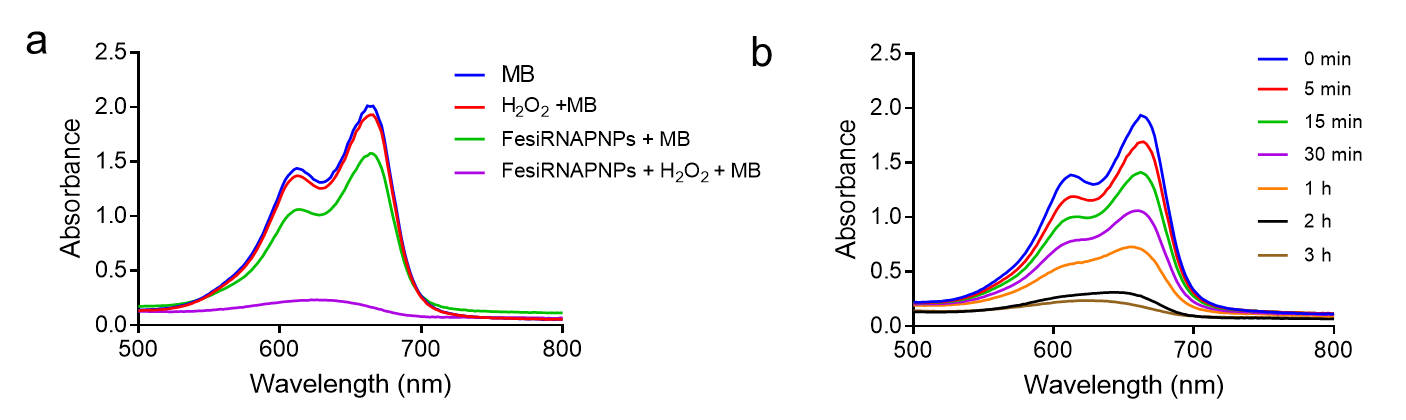


**Fig. S24.** (a) Degradation of MB triggered by H_2_O_2_, FesiRNAPNPs, and FesiRNAPNPs + H_2_O_2_ after 3 h of incubation. (b) Time-dependent UV-vis spectra of MB degradation triggered by FesiRNAPNPs + H_2_O_2_.


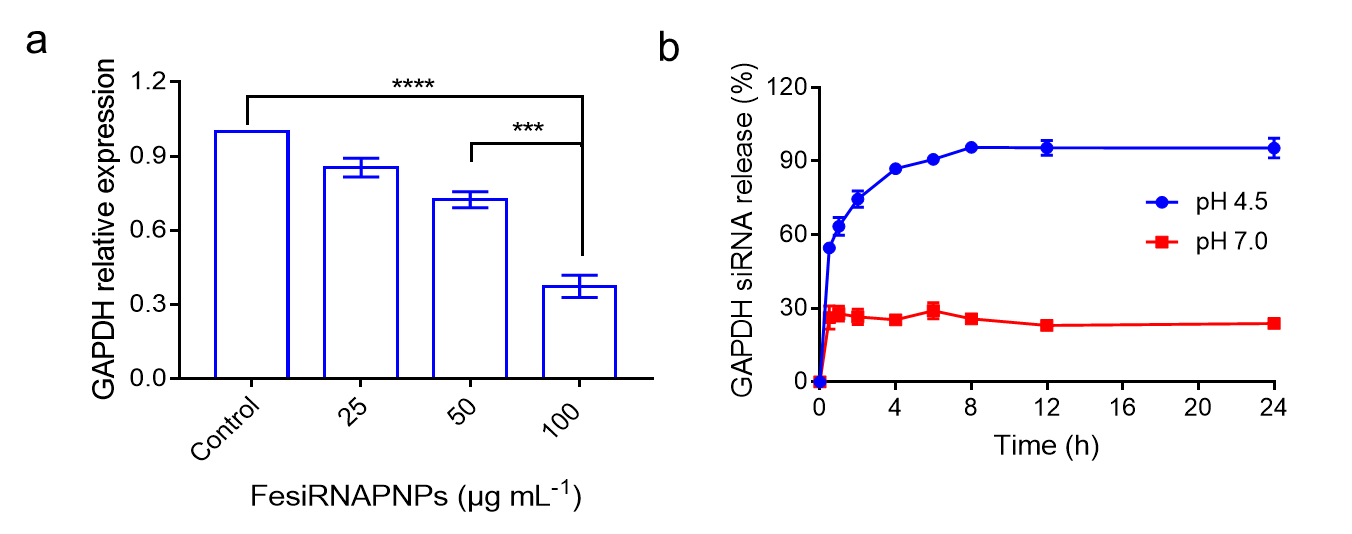


**Fig. S25.** (a) Real time-PCR analysis of the expression of GAPDH mRNA in CT26 cells after the treatment with FesiRNAPNPs. (b) Accumulated release profiles of GAPDH siRNA ions in various pH (7.0, 4.5)


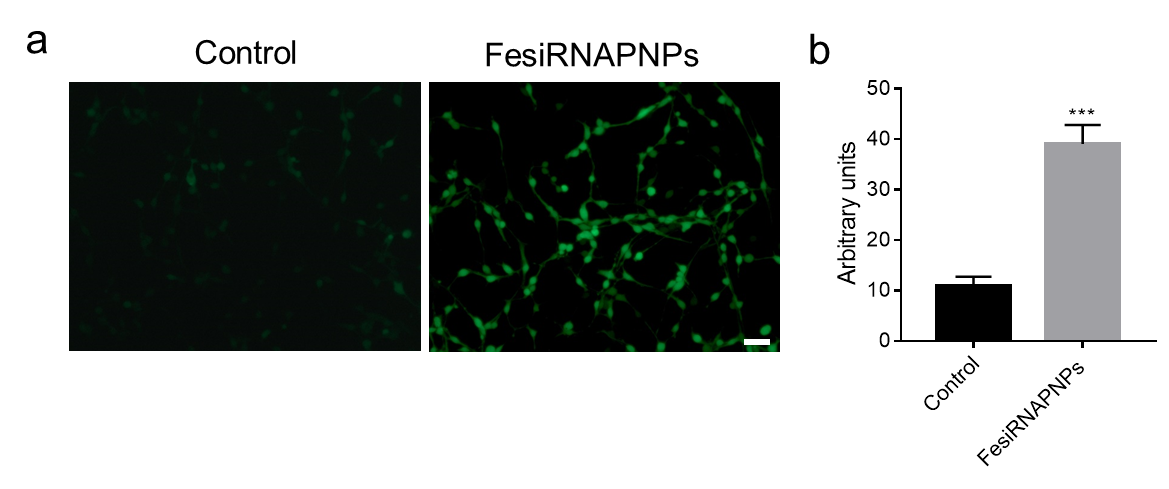


**Fig. S26.** (a) DCFH-DA assay of CT26 cells treated with FesiRNAPNPs (50 μg mL^-1^). Scale bar: 50 μm. (b) Corresponding ROS levels in CT26 cells after different exposure.


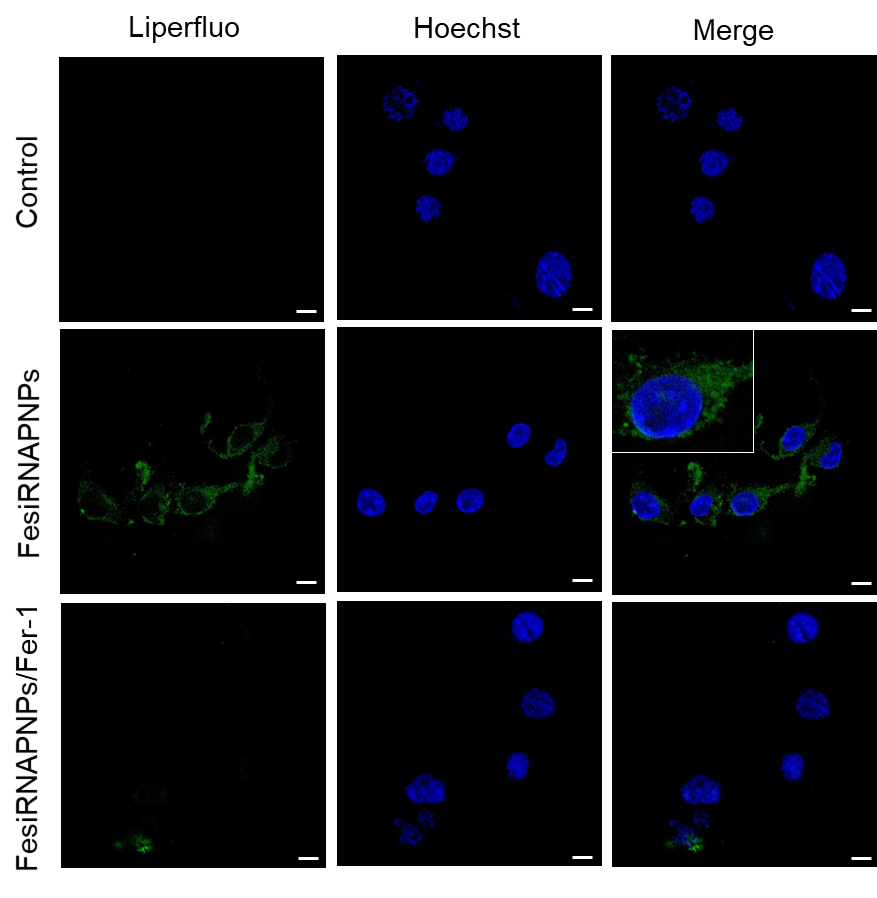


**Fig. S27.** CLSM images of Liperfluo staining in FesiRNAPNP-treated CT26 cells. Scale bar: 10 µm.

**Fig. S28.** Quantitative analysis for GPX4, NRF2, Keap1, P62, and GAPDH protein expression level in FeGPNPs and FesiRNAPNPs-treated CT26 cells.


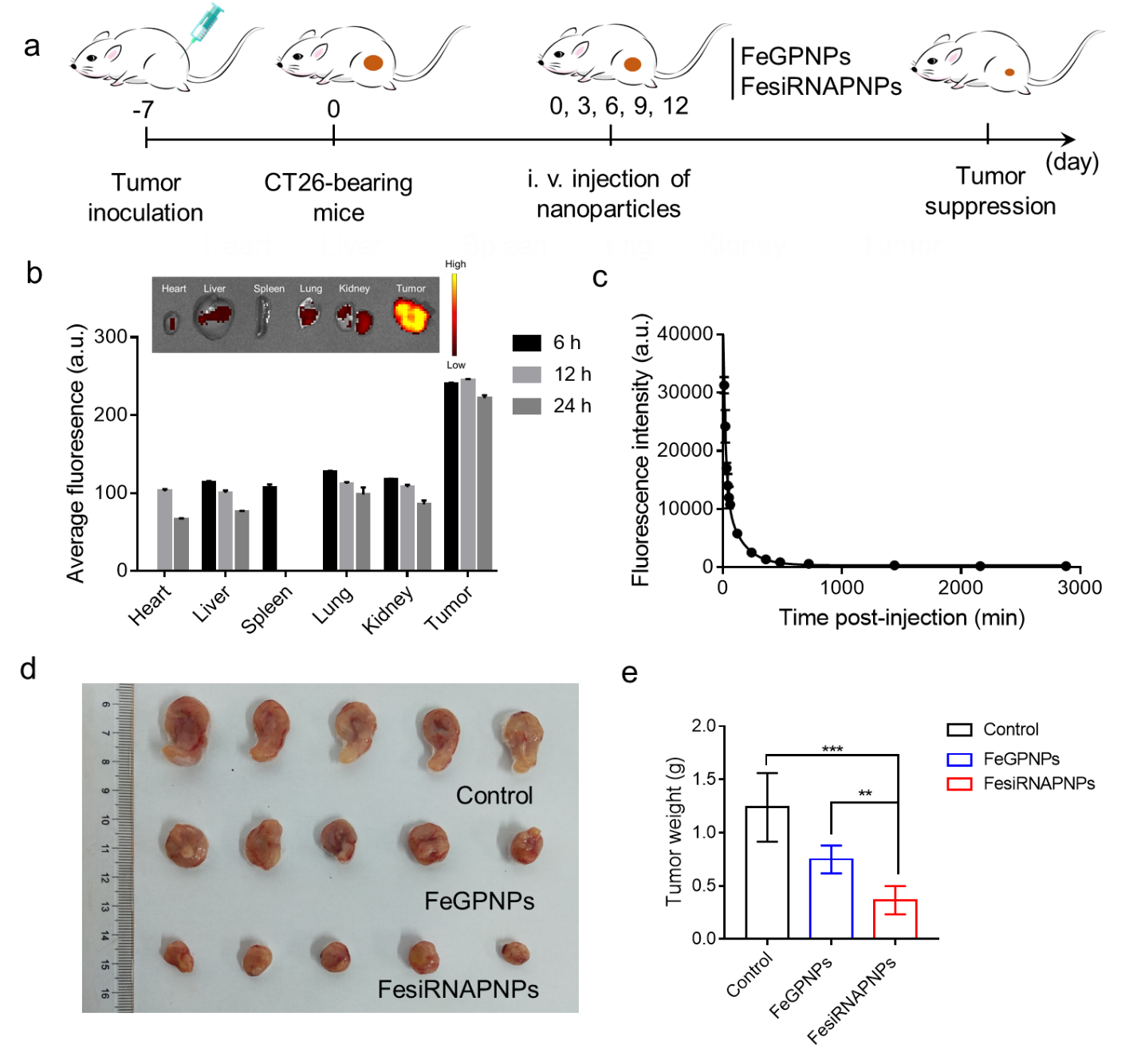


**Fig. S29.** (a) A scheme showing the experiment design. (b) Semi-quantitative analysis of ex vivo fluorescence images in major organs at different time points post i.v. injection of FesiRNAPNPs. Inset: Ex vivo fluorescence images of major organs and tumor dissected from CT26 tumor-bearing mice taken at 12 h post i.v. injection of FesiRNAPNPs. (c) Blood circulation curve of intravenously injected Ce6 labelled FesiRNAPNPs (Ce6- FesiRNAPNPs). Data are means ± SD (*n* = 3). (d) Digital pictures of tumors from the tumor-bearing mice after 15 days of therapy. (e) Average tumor mass excised from the tumor-bearing mice after treatment.

**Fig. S30.** Body weight changes in tumor-bearing after different treatments. Data are shown as means ± SD (*n* = 5).

**
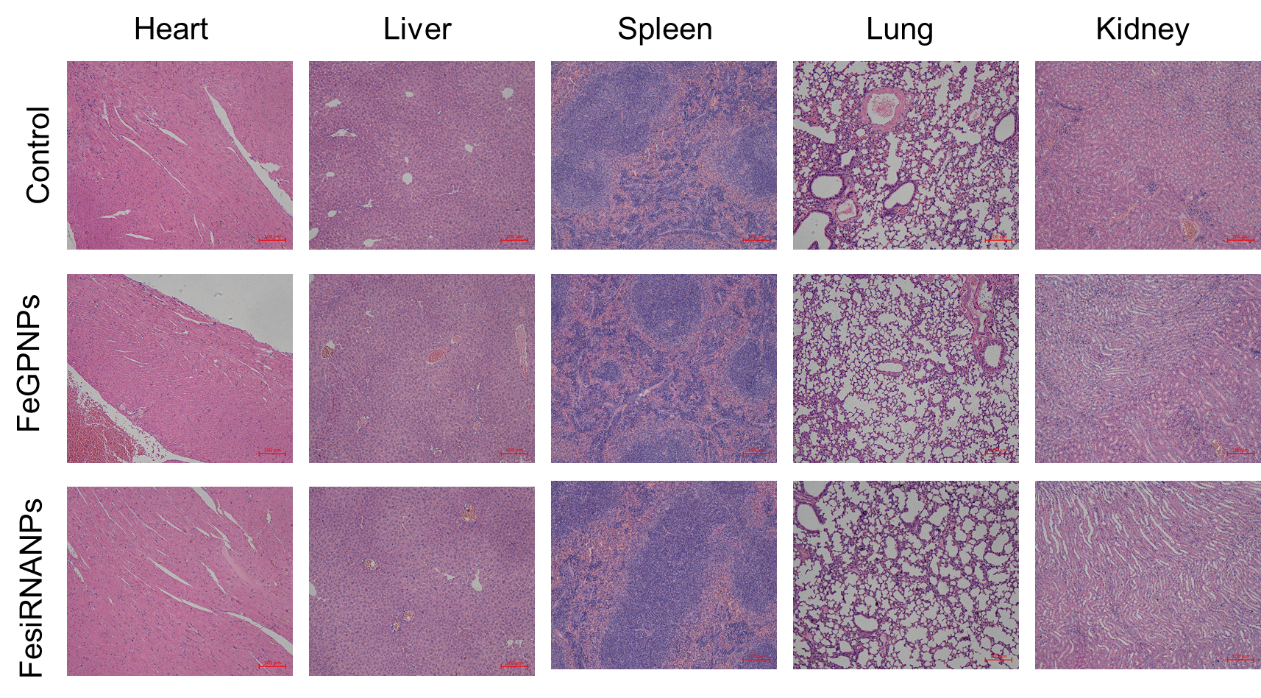
**

**Fig. S31.** H&E staining of the major organs (liver, spleen, heart, lung, and kidney) of tumor-bearing mice with i.v. injection of FeGPNPs and FesiRNAPNPs. Scale bar = 100 µm.

**Fig. S32.** Cytotoxicity of FesiRNAPNPs on normal cell line L02 for 24 h incubation.

**
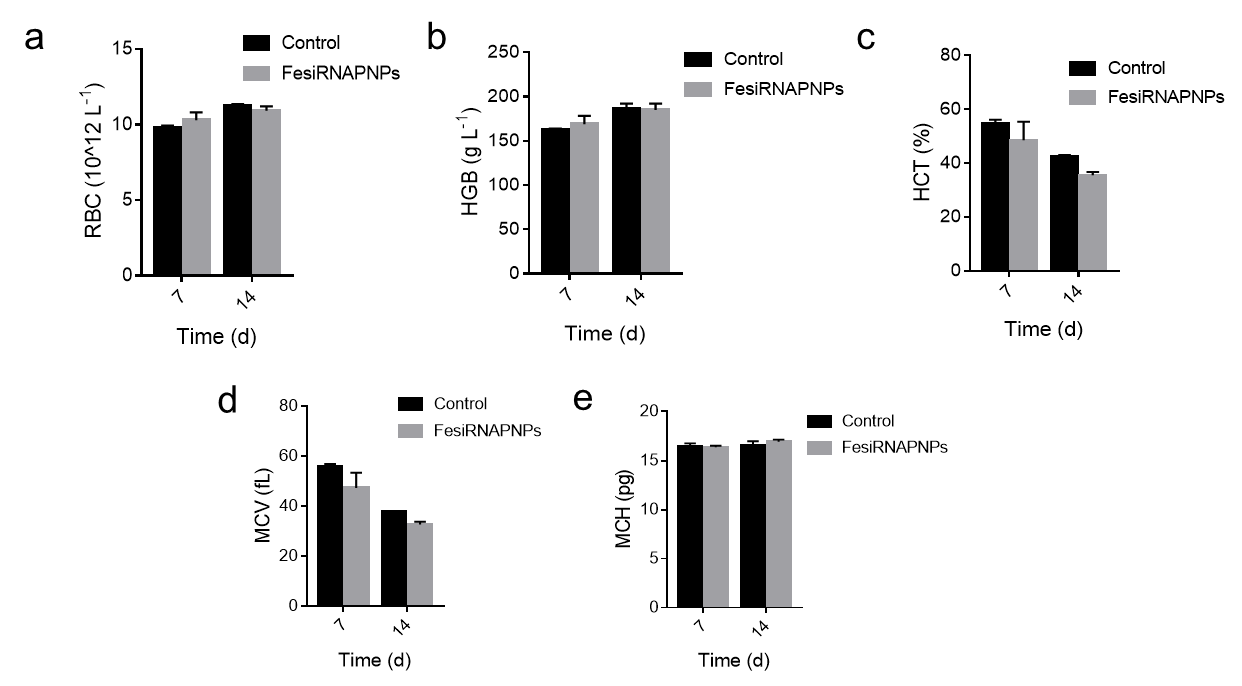
**

**Fig. S33.** Blood routine data with i.v. treatment of FesiRNAPNPs (25 mg kg^-1^) on day 7 and 14, including RBC, HGB, HCT, MCV, and MCH. Data are means ± SD (*n* = 3).

**
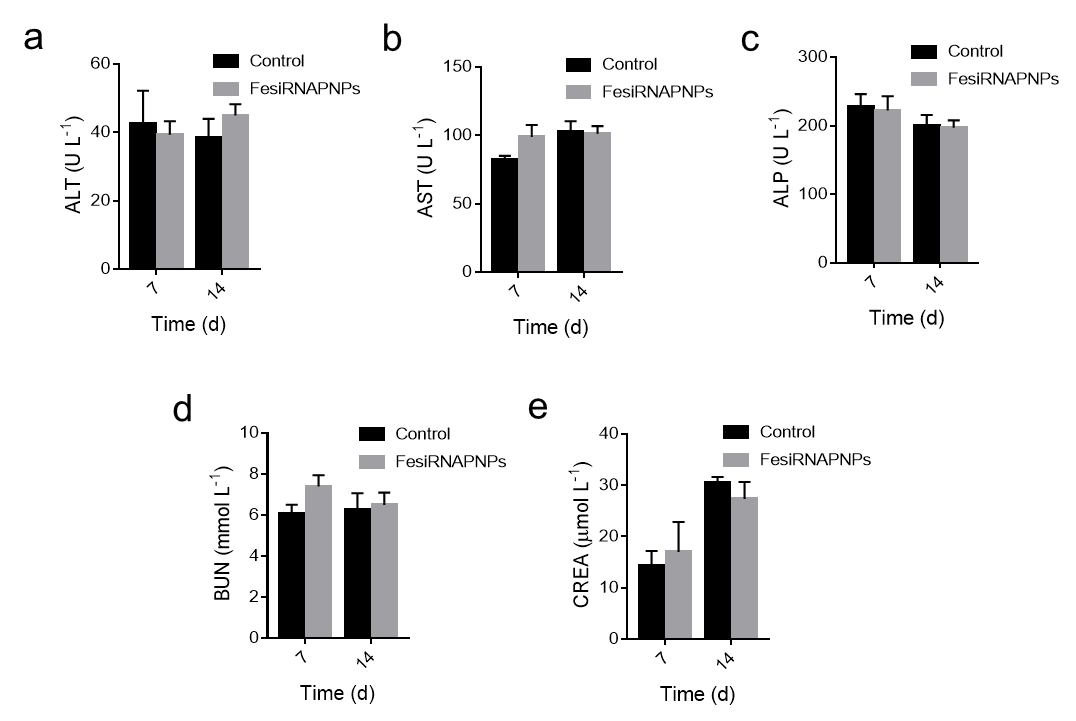
**

**Fig. S34.** Serum biochemistry data with i.v. treatment of FesiRNAPNPs (25 mg kg^-1^) on day 7 and 14, including liver function (ALP, ALT, and AST) and kidney function (CREA and BUN). Data are means ± SD (*n* = 3).

**
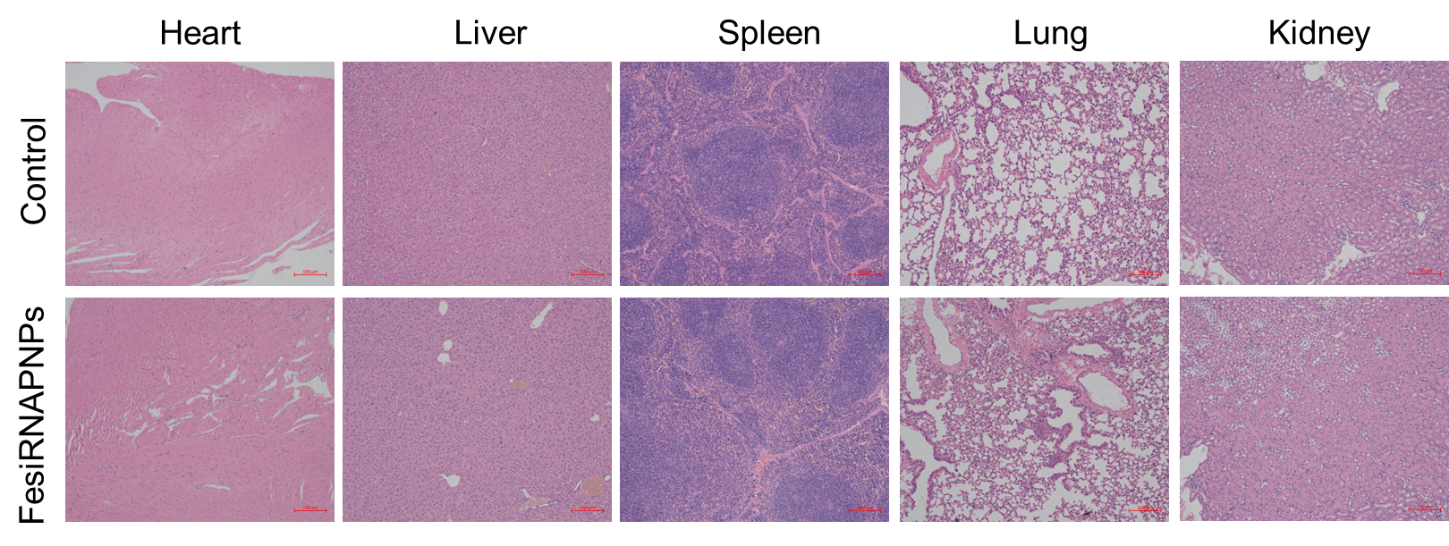
**

**Fig. S35.** H&E staining of the major organs (liver, spleen, heart, lung, and kidney) of healthy mice with i.v. injection of FesiRNAPNPs (25 mg kg^-1^). Scale bar = 100 µm.

**Table S1.** The kinetic parameters of FeGPNPs and FesiRNANPs. [E] was the concentration of nanoparticles, *K*_m_ is the Michaelis constant, *V*_max_ is the maximal reaction velocity is the catalytic constant.

**
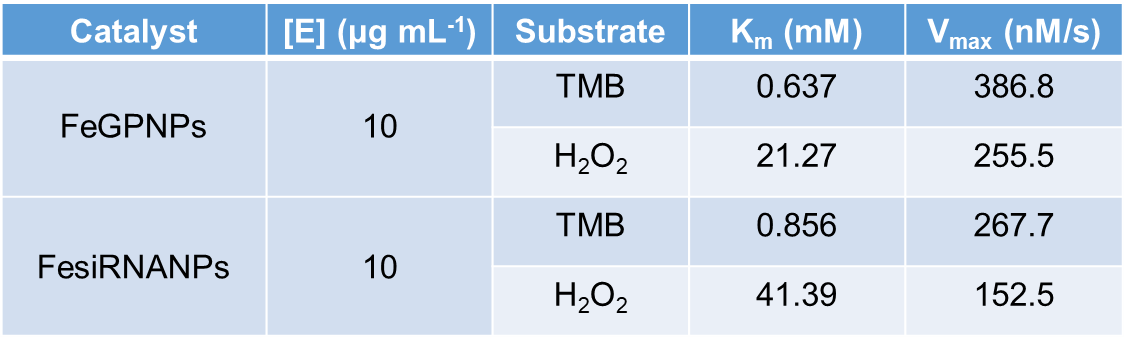
**
